# In Situ Growth Mechanism for High‐Quality Hybrid Perovskite Single‐Crystal Thin Films with High Area to Thickness Ratio: Looking for the Sweet Spot

**DOI:** 10.1002/advs.202104788

**Published:** 2022-03-08

**Authors:** Xiaobing Tang, Zhaojin Wang, Dan Wu, Zhenghui Wu, Zhenwei Ren, Ruxue Li, Pai Liu, Guanding Mei, Jiayun Sun, Jiahao Yu, Fankai Zheng, Wallace C. H. Choy, Rui Chen, Xiao Wei Sun, Fuqian Yang, Kai Wang

**Affiliations:** ^1^ Department of Electrical and Electronic Engineering Guangdong University Key Laboratory for Advanced Quantum Dot Displays and Lighting Guangdong‐Hong Kong‐Macao Joint Laboratory for Photonic‐Thermal‐Electrical Energy Materials and Devices Southern University of Science and Technology Shenzhen 518055 P. R. China; ^2^ Materials Program Department of Chemical and Materials Engineering University of Kentucky Lexington KY 40506 USA; ^3^ College of New Materials and New Energies Shenzhen Technology University Shenzhen 518118 P. R. China; ^4^ Key Laboratory of Energy Conversion and Storage Technologies (Southern University of Science and Technology) Ministry of Education Shenzhen 518055 P. R. China; ^5^ Department of Electrical and Electronic Engineering The University of Hong Kong Hong Kong P. R. China

**Keywords:** area to thickness ratio, crystal growth, hole‐transport layer, perovskite single‐crystal thin films

## Abstract

The development of in situ growth methods for the fabrication of high‐quality perovskite single‐crystal thin films (SCTFs) directly on hole‐transport layers (HTLs) to boost the performance of optoelectronic devices is critically important. However, the fabrication of large‐area high‐quality SCTFs with thin thickness still remains a significant challenge due to the elusive growth mechanism of this process. In this work, the influence of three key factors on in situ growth of high‐quality large‐size MAPbBr_3_ SCTFs on HTLs is investigated. An optimal “sweet spot” is determined: low interface energy between the precursor solution and substrate, a slow heating rate, and a moderate precursor solution concentration. As a result, the as‐obtained perovskite SCTFs with a thickness of 540 nm achieve a record area to thickness ratio of 1.94 × 10^4^ mm, a record X‐ray diffraction peak full width at half maximum of 0.017°, and an ultralong carrier lifetime of 1552 ns. These characteristics enable the as‐obtained perovskite SCTFs to exhibit a record carrier mobility of 141 cm^2^ V^−1^ s^−1^ and good long‐term structural stability over 360 days.

## Graphical abstract

In situ growth of single crystal thin films (SCTFs) on hole transport layers (HTLs) is of paramount importance for developing high‐performance optoelectronic devices. Here, hidden growth mechanism for MAPbBr_3_ SCTFs on HTLs is unraveled and high‐quality MAPbBr_3_ SCTFs with record largest area to thickness ratio are obtained through modulating three main factors: interface energy, precursor solution concentration, and heating rate.

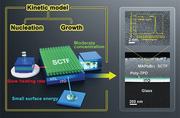

## 1 Introduction

Organic–inorganic hybrid perovskites have been widely recognized as promising materials for optoelectronic devices due to their high light‐absorption coefficients, tunable bandgaps, cost‐effective and facile preparation process.^[^ ^1^, ^2^, ^3^, ^4^, ^5^, ^6^, ^7^, ^8^, ^9^, ^10^, ^11^ ^]^ The perovskite and perovskite/silicon tandem solar cells with the highest power conversion efficiencies of 25.5%^[^ ^12^ ^]^ and 29%^[^ ^13^ ^]^ rival the performance of commercial polycrystalline silicon solar cells. Perovskite light‐emitting diodes (LEDs) with an external quantum efficiency over 20% approach the efficiencies of commercial organic LEDs.^[^ ^4^, ^5^, ^14^ ^]^ However, despite their rapid development, the active layers that perovskite polycrystalline thin films are composed of have unavoidable issues, including mobile ionic defects due to the low‐temperature solution process,^[^ ^15^, ^16^ ^]^ and high trap densities induced by grain boundaries.^[^ ^17^ ^]^

Compared with polycrystalline thin films, single‐crystal thin films (SCTFs) without grain boundaries are preferable for perovskite optoelectronic devices because the adoption of perovskite SCTFs can further improve performance due to their higher carrier mobility, longer carrier diffusion length, and higher stability.^[^ ^18^, ^19^, ^20^, ^21^, ^22^, ^23^, ^24^ ^]^ Generally, hybrid perovskite SCTFs can be grown on substrates, such as silicon, polyethylene terephthalate (PET), glass, indium tin oxide (ITO), fluorine‐doped tin oxide (FTO), polyimide (PI), SrTiO_3_,^[^ ^17^, ^25^, ^26^, ^27^, ^28^, ^29^ ^]^ and in solution.^[^ ^30^, ^31^, ^32^ ^]^ However, the perovskite SCTFs obtained from these reported methods cannot be directly used in optoelectronic device structures. This is because the majority of high‐performance solar cells, LEDs, and photodiodes based on hybrid perovskites possess vertical structures with active layers sandwiched between transport layers connected to respective electrodes. For perovskite SCTFs devices, such as solar cells, require carriers to be transferred from their active layer (perovskite SCTFs) through the charge‐transport layers (hole‐transport layers (HTLs) and electron‐transport layers (ETLs)) to their respective electrodes.^[^ ^33^, ^34^ ^]^ Due to the lack of proper synthesis methods for the in situ growth of perovskite SCTFs on widely used transport layers with underlying substrates, researchers need to adopt additional fabrication processes such as peeling the SCTFs from their initial grown substrate. These processes may cause unnecessary damage and/or introduce defects to these perovskite SCTFs. This jeopardizes the superiority of SCTFs and results in the deterioration of device performance.

To overcome these problems, the direct growth of high‐quality hybrid perovskite SCTFs on transport layers (e.g., HTLs) is required to limit transfer‐induced damage and defects and to improve the performance and integrity of these devices. Other transport layers (e.g., ETLs) can be fabricated on the opposite surface of perovskite SCTFs to form a ETL/perovskite/HTL structures for optoelectronic devices.^[^ ^35^, ^36^, ^37^ ^]^ Recently, the successful growth and morphological dependence of perovskite SCTFs on HTLs have been investigated and reported.^[^ ^35^, ^38^ ^]^ However, the thicknesses of the light‐harvesting perovskite layer of these SCTFs were 10–20 µm, which was too thick to extract charge carrier in an optoelectronic device. It should be pointed out that previous work has shown that film thicknesses of 400–800 nm can be used to successfully prepare high‐performance devices based on hybrid perovskites.^[^ ^1^, ^39^, ^40^ ^]^ On the other hand, optoelectronic devices with vertically sandwiched structures exhibit more excellent performance, benefiting from the perovskite active layer with larger area.^[^ ^41^ ^]^ Therefore, perovskite SCTFs with high area to thickness (ATT) ratio and thin thickness are expected to meet the rigorous material requirements for the fabrication of these devices. Chen et al.^[^ ^35^ ^]^ fabricated millimeter‐size (≈37.5 mm^2^ in area) SCTFs with a thickness of 10 µm and obtained a ATT of 3.75 × 10^3 ^mm. Rao et al.^[^ ^42^ ^]^ reported perovskite single crystals (6 × 8 mm^2^) with a thickness of 16 µm and an ATT of 3 × 10^3^ mm. Yang et al.^[^ ^28^ ^]^ achieved a thickness of 365 nm for perovskite SCTFs, with a side length and an ATT of ≈600 µm and 0.99 × 10^3^ mm, respectively. However, the growth of high‐quality hybrid MAPbBr_3_ SCTFs with high ATT ratios on HTLs still remains challenging because the growth of these crystals is isotropous in solution and the lateral growth of these films is confined once their thickness is restricted.^[^ ^26^, ^28^ ^]^ It should also be noted that the morphology and crystal quality of hybrid perovskite SCTFs on HTLs likely plays a critical role in determining the efficiency and stability of the devices.^[^ ^1^, ^43^ ^]^ Therefore, determining the elusive hidden mechanism for the nucleation and growth process of perovskite are highly desirable for obtaining high‐quality and high ATT ratio SCTFs.

In this work, in situ growth of high‐quality MAPbBr_3_ SCTFs with a high ATT ratio of 1.94 × 10^4^ mm on HTLs are realized for the first time. Through an in‐depth investigation into the mechanism of nucleation and growth of the SCTFs, we find a “sweet spot” with optimized growth factors for MAPbBr_3_ SCTFs with respect to the interface energy between the precursor solution and HTLs, the heating rate for crystals’ growth and the concentration of precursor solution. Millimeter‐scale MAPbBr_3_ SCTFs growing on poly(*N*,*N*″‐bis‐4‐butylphenyl‐*N*,*N*″‐bisphenyl)‐benzidine (poly‐TPD) HTLs layer are obtained. The (200) X‐ray diffraction (XRD) peak of these SCTFS exhibit a full width at half maximum (FWHM) of 0.017° which demonstrates their high crystallinity. And an ultra‐long average carrier lifetime of 1552 ns are achieved for the first time. Moreover, long‐term phase stability of 360 days and photoluminescence (PL) stability at temperatures up to 470 K are also observed. The high crystal quality and reliability of the MAPbBr_3_ SCTFs on HTLs demonstrate huge potential for future reliable perovskite optoelectronics.

## 2 Results and Discussion

**Figure 1:**
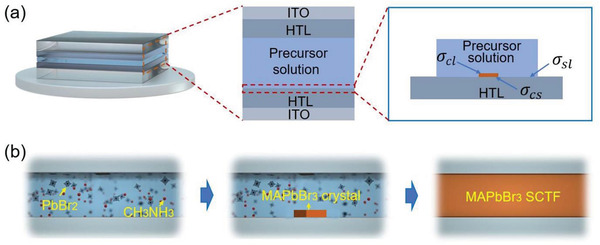
Schematic of the sandwich‐like structure for the a) nucleation and b) growth of a layer of MAPbBr_3_ SCTF from precursor solution.

According to the theory of thermodynamics, the synthesis of MAPbBr_3_ SCTFs was carried out in the structure as shown in **Figure** **1**a, where the precursors were sandwiched between two identical HTL‐coated ITO glass slides (see details in the Experimental Section). In this structure, the thicknesses of the MAPbBr_3_ SCTFs were tuned by adjusting the mass of the dead load on the top of the covering glass. There are two steps involving the formation and growth of MAPbBr_3_ SCTFs. The first one is the formation of an intermediate CH_3_NH_3_Br–PbBr_2_–DMF phase as shown in Equations (1) and (2)^[^ ^44^ ^]^

(1) $$\mathrm{PbB}r_{2}+\mathrm{DMF}\to \mathrm{PbB}r_{2}{\left(\mathrm{DMF}\right)}_{2}$$

(2) $$\mathrm{PbB}r_{2}{\left(\mathrm{DMF}\right)}_{2}+CH_{3}NH_{3}\mathrm{Br}\to CH_{3}NH_{3}\mathrm{Br}-\mathrm{PbB}r_{2}-\mathrm{DMF}$$

The second step is the conversion of the intermediate phase film to the perovskite phase via the extraction of DMF molecules during annealing, as described in Equation (3)^[^ ^44^ ^]^

(3) $$CH_{3}NH_{3}\mathrm{Br}-\mathrm{PbB}r_{2}-\mathrm{DM}F_{\left(l\right)}\to CH_{3}NH_{3}\mathrm{PbB}r_{3(s)}+\mathrm{DM}F_{\left(g\right)}$$

The sandwiched structure suppresses the growth of the intermediate phase film in the thickness direction and allows the intermediate phase film to laterally grow (Figure 1b).

The kinetic process for crystal growth consists of crystal nucleation and growth. Generally, nucleation on a foreign substrate is a heterogeneous nucleation process, without a gap between the substrate and the SCTFs as shown in the cross‐sectional view scanning transmission electron microscopy (STEM) image of the sample (Figure S1, Supporting Information). Because the prepared MAPbBr_3_ SCTFs are in a cubic phase, it is reasonable to deduce that the nucleus is formed also in cubic shape. For a cubic nucleus formed on a substrate, the nucleation rate (*J*_0_) can be calculated as^[^ ^45^ ^]^

(4) $$J_{0}=\omega^{*}\Gamma N^{*}$$

(5) $$N^{*}=N_{1}\exp\left(-\frac{\Delta G_{\mathrm{hetero}}^{*}}{RT}\right)$$

where N^*^ is the equilibrium concentration of critical nuclei, *N*_1_ is the concentration of monomers in the solution, ω* is the frequency of the attachment of monomers to the nucleus, and Γ is the Zeldovich factor. Moreover, the cubic crystal nuclei formed on a heterogeneous substrate is expressed as^[^ ^45^ ^]^

(6) $$\Delta G_{hetero}^{*}=\frac{32\sigma_{cl}^{3}V_{c}^{2}}{{\left(\Delta \mu \right)}^{2}}\frac{\left(\sigma_{cl}+\sigma_{cs}-\sigma_{sl}\right)}{2\sigma_{cl}}$$

(7) $$\Delta \mu =RT\ln\frac{C}{C_{0}}$$

where $\Delta G_{hetero}^{*}$ is the energy barrier for the formation of the nucleus, Δ*μ* is the change of chemical potential, depending on temperature *T* and the degree of supersaturation (Eq. (7)); *C* and *C_0_* are the solution concentration and equilibrium solution concentration, respectively, and *R* is the gas constant. *σ*_*cl*_,*σ*_*cs*_, and *σ*_*sl*_ are the interface energy between nucleus and precursor solution, the interface energy between nucleus and substrate and the interface energy between substrate and precursor solution, respectively (as shown in Figure 1a), *V*_c_ is a constant (molar volume of the crystal phase). The above Equations (4)–(7) demonstrate that the main factors of nucleation processes include temperature, concentration, and interface energies.

In general, a low crystal growth rate allows sufficient time for atoms to reach equilibrium sites, resulting in a high‐quality crystal. The relationship between the total growth rate (*R*_T_) and the concentration in a supersaturated solution can be expressed as^[^ ^46^ ^]^

(8) $$R_{T}=-\frac{d\Delta C}{dt}$$

where ΔC is the supersaturation degree of the solute in the solution. For an isothermal process, Equation (8) is rewritten as

(9) $$R=-\frac{d\Delta C}{dT}\frac{dT}{dt}$$

The solute solubility of MAPbBr_3_ decreases with the increase of temperature (Figure S2, Supporting Information), so as the concentration of the solute. Therefore, a higher initial concentration of the precursor solution and a higher temperature contribute a larger supersaturation. However, reaching supersaturation too rapidly does not favor the growth of high‐quality crystals. Thus, an appropriate precursor concentration is preferable. More importantly, the variation rate of the concentration ($\frac{d\Delta C}{dT}$) decrease as the temperature increases. According to Equation (9), the crystal growth rate is proportional to the ramp rate of temperature, which means a lower heating rate can result in a lower crystal growth rate.

According to the nucleation analysis above, the interface energy between precursor solution and substrate plays an important role in determining the nucleation behavior of MAPbBr_3_ SCTFs. Figure S3a–c (Supporting Information) presents optical images of the MAPbBr_3_ SCTFs formed on three different substrates, which are ITO covered with HTLs (poly‐TPD/ITO, poly(9,9'‐dioctylfluorene‐co‐bis‐N,N'‐(4‐butylphenyl)‐diphenylamine) (TFB)/ITO, and poly(3,4‐ethylenedioxythiophene) polystyrene sulfonate (PEDOT:PSS)/ITO), at a heating rate of 10 °C h^−1^ under the dead load of 2 kg. The XRD patterns in Figure S3d (Supporting Information) show that all the SCTFs are single crystalline with a cubic phase. The thickness of the MAPbBr_3_ SCTF grown on ploy‐TPD is ≈540 nm (Figure S4, Supporting Information). This demonstrates that the formed MAPbBr_3_ SCTFs are presented in different morphologies, depending on the substrate. For the poly‐TPD/ITO and TFB/ITO substrates, MAPbBr_3_ SCTFs with relatively regular shapes were grown, however, for PEDOT:PSS/ITO substrate, MAPbBr_3_ SCTFs in dendritic shape were grown.

We calculated the surface energies of the three substrates and the interface energies between the precursor solutions and the three substrates with different HTLs. Precursor solution, deionized (DI) water, and dimethyl sulfoxide (DMSO) were used in the measurement of the contact angles of precursor solution, (DI) water and DMSO on three HTLs covered ITO substrates (Figures S5a and S6, Supporting Information) and the results are summarized in **Table** **1** and Tables S1–S3 (Supporting Information). It is evident that the contact angles between the precursor solution and HTLs are in a range of 20.8° to 52.2°, suggesting that PEDOT:PSS are more favorable for the wetting of the precursor solution than Poly‐TPD and TFB. The difference in these contact angles can be attributed to the difference in the interface energies.

**Table 1:** Interface energy between the precursor solution and the substrate

	*σ*_sv_ [mN m^−1^]	*σ*_lv_ [mN m^−1^]	*θ* [°]	Cos *θ*	*σ*_sl_ [mN m^−1^]
Poly‐TPD	53.99	37.19	52.2	0.61	31.30
TFB	47.47	37.19	44.0	0.72	20.69
PEDOT:PSS	90.37	37.19	20.8	0.93	55.78

The correlation between surface energy and interface energy for the contact angle (*θ*) between a liquid and a solid substrate can be expressed as^[^ ^47^ ^]^

(10) $$\begin{array}{ccc} \frac{1+\cos\theta }{2} & = & \frac{{\left(\sigma_{sv}^{d}\sigma_{lv}^{d}\right)}^{1/2}}{\sigma_{lv}}+\frac{{\left(\sigma_{sv}^{p}\sigma_{lv}^{p}\right)}^{1/2}}{\sigma_{lv}}\;\mathrm{and}\;\sigma_{sl}={\left(\sqrt{\sigma_{sv}^{d}}-\sqrt{\sigma_{lv}^{d}}\right)}^{2}\\ & & +\;{\left(\sqrt{\sigma_{sv}^{p}}-\sqrt{\sigma_{lv}^{p}}\right)}^{2} \end{array}$$

where *σ*_sv_, *σ*_lv_, and *σ*_sl_ are the surface energy of a solid substrate and a liquid, and the interface energy between the substrate and the liquid, respectively, and the superscripts of “d” and “p” represent the dispersive and polar components of the surface energy, respectively. Using Equation (10), the measured contact angles and the numerical values available in the literature,^[^ ^30^ ^]^ we obtain surface energies of 53.99, 47.47 and 90.37 mN m^−1^ for poly‐TPD, TFB and PEDOT:PSS, respectively (see details in Tables S1‐S3 in the Supporting Information).

Note that no deformation was observed on the three different HTLs when performing the contact angle measurements. Therefore, the HTLs are considered to be rigid enough to apply the Young's equation^[^ ^48^ ^]^ to calculate the interface energies between the precursor solution and the HTLs. The calculated interface energies are listed in Table 1 and presented in Figure S5b (Supporting Information). It is evident that the interface energy between the precursor solution and the PEDOT:PSS film is the largest indicating the strongest interaction. In contrast, the interface energies between the precursor solution and the Poly‐TPD or TFB are smaller indicating weaker interaction. A strong interaction is more favorable for the nucleation of MAPbBr_3_ compared with a weak interaction, as demonstrated in Figure S7 (Supporting Information). However, the strong interaction between the monomers and the PEDOT:PSS limits the migration/diffusion of monomers on the surface of the PEDOT:PSS, leading to lateral growth of MAPbBr_3_ islands and the formation of dendrites rather than large films, as shown in Movie S1 (Supporting Information) and Figure S3c (Supporting Information). The growth of SCTFs was also studied on NiO_x_/ITO (81.79 mN m^−1^), ZnMgO/ITO (69.67 mN m^−1^), and SnO_2_/ITO (90.05 mN m^−1^) substrates (Figure S8 and Table S4, Supporting Information). All these SCTFs are dendrites, suggesting the important role of the interface energy between the precursor solution and the substrate in controlling the nucleation and morphology of MAPbBr_3_ films/islands.

However, weaker interaction leads to easier migration/diffusion of monomers in solution. Therefore, on the surface of the poly‐TPD, the lateral growth of the MAPbBr_3_ films with large sizes easily occurs (Figure S3a, Supporting Information).

From the perspective of thermodynamics, the driving force for the nucleation and growth of MAPbBr_3_ SCTFs is the difference of the Gibbs free energies, Δ*G*, between the precursor solution and the MAPbBr_3_ crystals as

(11) $$\Delta G=G_{C}-G_{P}$$

where *G*_C_ and *G*_P_ are the Gibbs free energies of the MAPbBr_3_ crystals and the precursor solution, respectively. Under the conditions of constant temperature and pressure, it needs Δ*G* < 0 for the nucleation and growth to occur. The difference of the molar Gibbs free energies consists of the contribution from the change of the Gibbs free energy due to the phase change and the interface energy between the MAPbBr_3_ crystal and the solution. For the nucleation in an infinite system, the contribution of the entropy change is negligible, while one needs to incorporate the contribution of the entropy change in the calculate of the Gibbs free energy for a finite system.^[^ ^49^ ^]^ The system for nucleation in present work is approximately infinite, so the entropy change is negligible. Note that a supersaturated solution is needed for the nucleation and growth of any new phases, including MAPbBr_3_ SCTFs.

Consider the nucleation of a MAPbBr_3_ crystal nucleus in an n‐sided (n = 4 in this work), regular polygon in the sandwiched structure shown in Figure 1a. Assuming that the thickness of the crystal film is *h* and the side‐length is *a*, and only one surface of the MAPbBr_3_ SCTF is in contact with the HTL (Figure 1b), the difference of the Gibbs free energies, Δ*G*,^[^ ^50^ ^]^ can be expressed as

(12) $$\Delta G=\frac{na^{2}h}{4}\cot\frac{\pi }{n}\Delta G_{V}+\frac{na^{2}}{4}\cot\frac{\pi }{n}\left(\sigma_{cl}+\sigma_{cs}-\sigma_{sl}\right)+nah\sigma_{cl}$$

Under the condition that the volume of the MAPbBr_3_ crystal nucleus is constant, the minimum interface energy between MAPbBr_3_ SCTF and HTL yields

(13) $$h=\frac{a}{2}\frac{\left(\sigma_{cl}+\sigma_{cs}-\sigma_{sl}\right)}{\sigma_{cl}}\cot\frac{\pi }{n}$$

Substituting Equation (13) in Equation (12) and taking derivative with respect to *a*, we obtain the critical side‐length and thickness of a nucleus, *a*_c_ and *h*_c_, as

(14) $$a_{c}=-\frac{4\sigma_{cl}}{\Delta G_{V}}\tan\frac{\pi }{n}$$

(15) $$h_{c}=-\frac{2(\sigma_{cl}+\sigma_{cs}-\sigma_{sl})}{\Delta G_{V}}$$

It is evident that the critical side‐length of the MAPbBr_3_ nucleus is only dependent on the interface energy between the MAPbBr_3_ crystal nucleus and the precursor solution, and the critical thickness of the MAPbBr_3_ crystal nucleus is dependent on (*σ*_cl_ + *σ*_cs_ − *σ*_sl_). That is to say that the critical side‐length size is independent of the HTL used in the sandwiched structure. Note that for a slow constant heating rate, the system can be considered as a quasi‐thermal equilibrium process.

Using the density functional theory (DFT), we analyzed the interaction between HTLs (poly‐TPD, TFB, PEDOT:PSS) and a MAPbBr_3_ SCTF, as shown in Figure S9 (Supporting Information). The calculations are based on the interaction between HTLs and MABr‐terminated MAPbBr_3_ surfaces because the MABr holds minimum energy surface terminations for MAPbBr_3_.^[^ ^51^ ^]^ The calculation results show that the corresponding adsorption energies (*E*_ads_) for MAPbBr_3_/poly‐TPD, MAPbBr_3_/TFB and MAPbBr_3_/PEDOT:PSS are −0.583 eV (−13.4 kcal mol^−1^), −0.972 eV (−22.4 kcal mol^−1^), and −1.473 eV (−34.0 kcal mol^−1^), respectively, suggesting that it is a weak hydrogen bonds interaction between HTLs and a MAPbBr_3_ single‐crystal film. In addition, XRD (Figure S10, Supporting Information) analysis indicates that the HTLs polymers are all amorphous. Fourier transform infrared spectroscopy (FTIR) (Figure S11, Supporting Information) shows that the peak positions remain unchanged before and after the formation of MAPbBr_3_ SCTFs on the HTLs, suggesting there is no obvious chemical interaction, further proving that the interaction is hydrogen bonding. The results of DFT, XRD, and FTIR suggest that the growth of MAPbBr_3_ SCTFs on the HTLs is based on hydrogen bond interactions. According to the optical micrographs in Figure S3 (Supporting Information) and the interface energies in Table 1, it can be concluded that the Poly‐TPD and TFB films are the most suitable HTLs for the growth of MAPbBr_3_ SCTFs for the conditions used in this work. The following analysis is focused on the MAPbBr_3_ SCTFs grown on the poly‐TPD/ITO.

A smaller crystal growth rate can produce higher‐quality crystals since a smaller growth rate is beneficial for monomer to reach equilibrium sites. Figure S12 (Supporting Information) depicts optical micrographs of the MAPbBr_3_ SCTFs grown on poly‐TPD/ITO at three different heating rates (10, 5, and 2 °C h^−1^), which lead to the MAPbBr_3_ SCTFs surface areas of ≈0.2, ≈0.4, and ≈10.5 mm^2^, respectively. The corresponding surface‐root‐mean‐square roughness (RMS) are ≈0.624, ≈0.601, and ≈0.598 nm for the SCTFs grown at heating rates of 10, 5, and 2 °C h^−1^, respectively. This indicates decreasing the heating rate enables the growth of large‐area MAPbBr_3_ SCTFs with better surface quality. Similar results can also be obtained on the other two HTLs (Figure S13, Supporting Information). To achieve a smaller scale (e.g., nanometer scale) morphologies of MAPbBr_3_ SCTFs at various heating rate, a set of top‐view scanning electron microscope (SEM) images were obtained, as shown in Figure S14 (Supporting Information). Figure S14a–c (Supporting Information) are presents the SCTFs prepared at heating rates of 10, 5, and 2 °C h^−1^, respectively. The extremely flat and smooth surface verifies the high quality of SCTFs grown under all the three heating rates. This behavior can be attributed to monomers in the precursor solution at a low heating rate have relatively enough time to reach equilibrium sites on the edge of the MAPbBr_3_ single‐crystal films during growth, leading to the growth of large high‐quality MAPbBr_3_ SCTFs.

**Figure 2:**
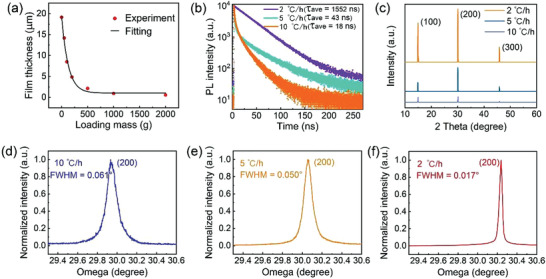
a) Variation of the thickness of the MAPbBr_3_ SCTFs with the dead load on the sandwiched structures, b) TRPL decay transients of the MAPbBr_3_ SCTFs under 405 nm excitation, c) XRD patterns for MAPbBr_3_ SCTFs prepared under various heating rate at room temperature (25 °C), and d–f) high‐resolution XRD rocking curves of the (200) diffraction peaks of the MAPbBr_3_ SCTFs at a heating rate of d) 10 °C h^−1^, e) 5 °C h^−1^, and f) 2 °C h^−1^.

**Figure** **2**a shows the variation in the thickness of the MAPbBr_3_ SCTFs with the dead loads applied to the sandwiched structure grown at a heating rate of 2 °C h^−1^. The thickness of the MAPbBr_3_ SCTFs decreases with the increase of the dead load, reaching ≈540 nm at a dead load of 2 kg, suggesting that a dead load can be used to tailor the thickness of the MAPbBr_3_ SCTFs. It is evident from the Equations (4)–(7) that the nucleation rate of crystals is only related to three factors: growth temperature, precursor solution concentration, and the interface energy between two phases, and pressure does not have a significant effect on nucleation rate.^[^ ^45^ ^]^ According to the crystal growth rate equations (Equations (8) and (9)), on a same substrate (surface energy is fixed), the crystal growth rate only depends on the heating rate and the precursor solution concentration.^[^ ^46^ ^]^ The effect of pressure on the crystal growth is negligible. From the theory of crystal nucleation and growth, it can be concluded that the pressure has insignificant effect on the crystal nucleation and growth process, and also on the quality of the prepared crystal films. However, in the process of preparing SCTFs by the space confinement method, the pressure can change the space, thereby adjusting the thickness of the thin films.^[^ ^17^, ^28^ ^]^ Next, we performed XRD test on SCTFs prepared under various dead loads. The result is shown in Figure S15 (Supporting Information). All the XRD patterns of MAPbBr_3_ SCTFs prepared under a dead load of 0 kg, 1 kg, and 2 kg have periodic diffraction peaks assigned to (100), (200), and (300) planes. The comparable FWHM of the diffraction peaks of SCTFs obtained under three dead loads indicate the three types of SCTFs have an insignificant difference in crystallinity. This is in good agreement with the expected trend based on crystal nucleation and growth theory.The XRD results suggest that within a certain pressure range, the pressure has little effect on pervoskite crystals nucleation and growth, so it has little effect on the quality of the SCTFs.^[^ ^17^, ^28^ ^]^ Nevertheless, if the pressure on the top substrate increases infinitely and the space between two substrates becomes smaller, it will be difficult for the solvent in the precursor to volatilize and the longitudinal growth of the SCTFs will be confined, resulting in a smaller SCTFs area.^[^ ^17^, ^28^ ^]^ Therefore, this situation is not discussed in detail in the present work.

The defect states concentration of the SCTFs was characterized by time‐resolved photoluminescence (TRPL) (Figure 2b). The process of decay has a long lifetime component and a short lifetime component. The electrons in semiconductors are excited to an excited state when semiconductors absorb photons and the excess excited electrons return back to ground state with a recombination with holes.^[^ ^52^ ^]^ A recombination process generally can be radiative or nonradiative types.^[^ ^52^, ^53^ ^]^ During a radiative recombination process, photons are emitted after the holes and electrons recombine. In contrast, during a nonradiative recombination process no photon emission occurs due to the trap‐induced carrier recombination.^[^ ^52^, ^53^ ^]^ During this photophysical process the radiative carrier lifetime and nonradiative carrier lifetime can be obtained by fitting the decay curves using a double exponential function^[^ ^54^ ^]^

(16) $$I_{pl}=A_{1}\exp\left(-t/\tau_{1}\right)+A_{2}\exp\left(-t/\tau_{2}\right)$$

where *I*_pl_ refers to PL intensity, *τ*_1_ and *τ*_2_ are radiative carrier lifetime and nonradiative carrier lifetime, respectively, A_1_ and A_2_ are the fractional amplitude (A_1_+A_2_= 1) for *τ*_1_ and *τ*_2,_ respectively. An average carrier lifetime (*τ*_*ave*_) is determined by^[^ ^54^ ^]^

(17) $$\tau_{ave}=A_{1}\tau_{1}+A_{2}\tau_{2}$$

Table S5 (Supporting Information) summarizes long‐lived radiative component (*τ*_1_), short‐lived nonradiative component (*τ*_2_), and average carrier lifetime (*τ*_*ave*_) of the MAPbBr_3_ SCTFs synthesized under various heating rates. It is evident that the average carrier lifetime (*τ*_*ave*_) (Figure 2b) increases with the decrease of the heating rate. This is because slower heating rate results in slower crystal growth rate, which leads to higher the crystal quality and lower the defect state concentration. The longest average carrier lifetime is 1552 ns for the MAPbBr_3_ SCTFs grown at the heating rate of 2 °C h^−1^, which is much longer than those grown at the heating rate of 5 and 10 °C h^−1^. This is about twice as high as the average carrier lifetime of 815 ns reported for bulk single crystals by Liu et al..^[^ ^23^ ^]^ Such a result reveals that small heating rate favors the growth of high‐quality MAPbBr_3_ SCTFs with a low trap density.

The crystallinity of the SCTFs was characterized by XRD. As shown in Figure 2c, the XRD patterns of the MAPbBr_3_ SCTFs shows periodic diffraction peaks at 14.93°, 30.08°, and 45.83° corresponding to the (100), (200) and (300) crystal planes, respectively. This verifies cubic structure (space group *Pm*3*m*) and strong single crystallinity of the MAPbBr_3_ SCTFs without any residual raw materials (precursor powders) (see XRD results in Figure S16 in the Supporting Information), which is consistent with the other reports.^[^ ^23^ ^]^ XRD patterns shown in Figure S17 (Supporting Information) indicate high‐quality MAPbI_3_, (PEA)_2_PbI_4_, (PEA)_2_PbBr_4_ SCTFs grown on poly‐TPD at 2 °C h^−1^. This suggests the method used in this work is applicable for different materials in the perovskite family. In the XRD pattern of MAPbI_3_ SCTFs, the diffraction peaks at 31°, 44°, 57° correspond to (220), (330), (440) crystal planes, respectively. This is in good agreement with previous reports.^[^ ^55^ ^]^ Especially, more details of characterizations in terms of MAPbI_3_ SCTFs are discussed herein. Figure S18a (Supporting Information) represents a top‐view SEM image of a MAPbI_3_ SCTF grown on a Poly‐TPD/ITO/glass substrate. It is clear that no obvious grain boundaries are observed on the MAPbI_3_ thin film, which verifies the single‐crystalline property of the as‐synthesized MAPbI_3_ thin film.^[^ ^35^, ^36^, ^37^ ^]^ Figure S18b,c (Supporting Information) illustrate PL and absorption spectra of a single MAPbI_3_ SCTF with PL peak and absorption peak of 780 and 762 nm, respectively, indicating a Stokes shift of 18 nm. The clear cutoff band edge of the absorption spectrum demonstrates a low exciton binding energy, further indicating MAPbI_3_ films are single crystalline films with few in‐gap defect states.^[^ ^53^, ^56^ ^]^ Compared with reported polycrystalline films with a PL peak (770 nm) and absorption peak (750 nm), the PL and absorption peaks of MAPbI_3_ thin film in the present work experiences red shift of 10 and 12 nm, respectively.^[^ ^57^ ^]^ Both PL peak and absorption peak are red‐shifted because of the long‐range ordered arrangement of atoms and less defect states concentration in the crystal, which also proves that the MAPbI_3_ film in the present work is a single crystal film with high crystalline quality.^[^ ^53^, ^56^ ^]^ Therefore, the characterizations of XRD, SEM, PL and absorption together prove that the MAPbI_3_ film is single‐crystalline. The high‐resolution XRD rocking curves of the MAPbBr_3_ SCTFs are presented in Figure 2d–f. The (200) peaks are centered at 29.938°, 30.238°, and 30.056° for the MAPbBr_3_ SCTFs grown at 10, 5, and 2 °C h^−1^, respectively, with a corresponding FWHM of the diffraction peaks of 0.061°, 0.050°, and 0.017° for respective sample. Interestingly, the FWHM of 0.017° for the MAPbBr_3_ SCTFs grown at 2 °C h^−1^ is even smaller than that of the bulk single crystals of 0.021°,^[^ ^23^ ^]^ suggesting extremely high quality and excellent crystallinity of the as‐synthesized MAPbBr_3_ SCTFs. The small FWHM of 0.017° and long average carrier lifetime (1552 ns) represents a notable breakthrough in achieving high crystallinity and restricting defects for the of MAPbBr_3_ SCTFs (**Table** **2**).

**Table 2:** Comparison between the best reported MAPbBr_3_ perovskite single crystal thin films and this work

Materials	*τ*_ave_ [ns]	FWHM of XRD peak [°]	Area [mm^2^]	Thickness [mm]	Ratio of area to thickness [mm]	Ref.
MAPbBr_3_	262	0.079	48	1.6 × 10^−2^	3 × 10^3^	^[^ ^42^ ^]^
MAPbBr_3_	–	–	37.5	1.0 × 10^−2^	3.75 × 10^3^	^[^ ^35^ ^]^
MAPbBr_3_	242	0.05	0.36	3.65 × 10^−4^	0.99 × 10^3^	^[^ ^28^ ^]^
MAPbBr_3_	< 93	–	1.69 × 10^−4^	1.1 × 10^−3^	0.154	^[^ ^59^ ^]^
MAPbBr_3_	< 391	–	1.4	6.05 × 10^−4^	2.3 × 10^3^	^[^ ^26^ ^]^
MAPbBr_3_	1552	0.017	10.5	5.4 × 10^−4^	1.94 × 10^4^	This work

We also note that increasing the heating rate leads to the left‐handed shift (decrease of omega) of the (200) peaks, suggesting an increase of the lattice constant of SCTFs. The increase of lattice constant of SCTFs can introduce mechanical deformation to the SCTFs, resulting in an inferior crystallinity. This corresponds to the results of TRPL in Figure 2b. The PL spectra (Figure S19, Supporting Information) shows that the PL peaks of samples grown under large heating rate experience a slight blue shift, suggesting the effect of the lattice constant on the energy gap of the crystal. The blue shift of PL peaks indicates an increase of the bandgap of SCTFs under an increasing heating rate.^[^ ^53^, ^56^ ^]^ Combining the XRD results (Figure 2d–f) it was concluded that increasing heating rates give rise to a larger lattice constant and bandgap of MAPbBr_3_ SCTFs.^[^ ^53^, ^56^ ^]^

**Figure 3:**
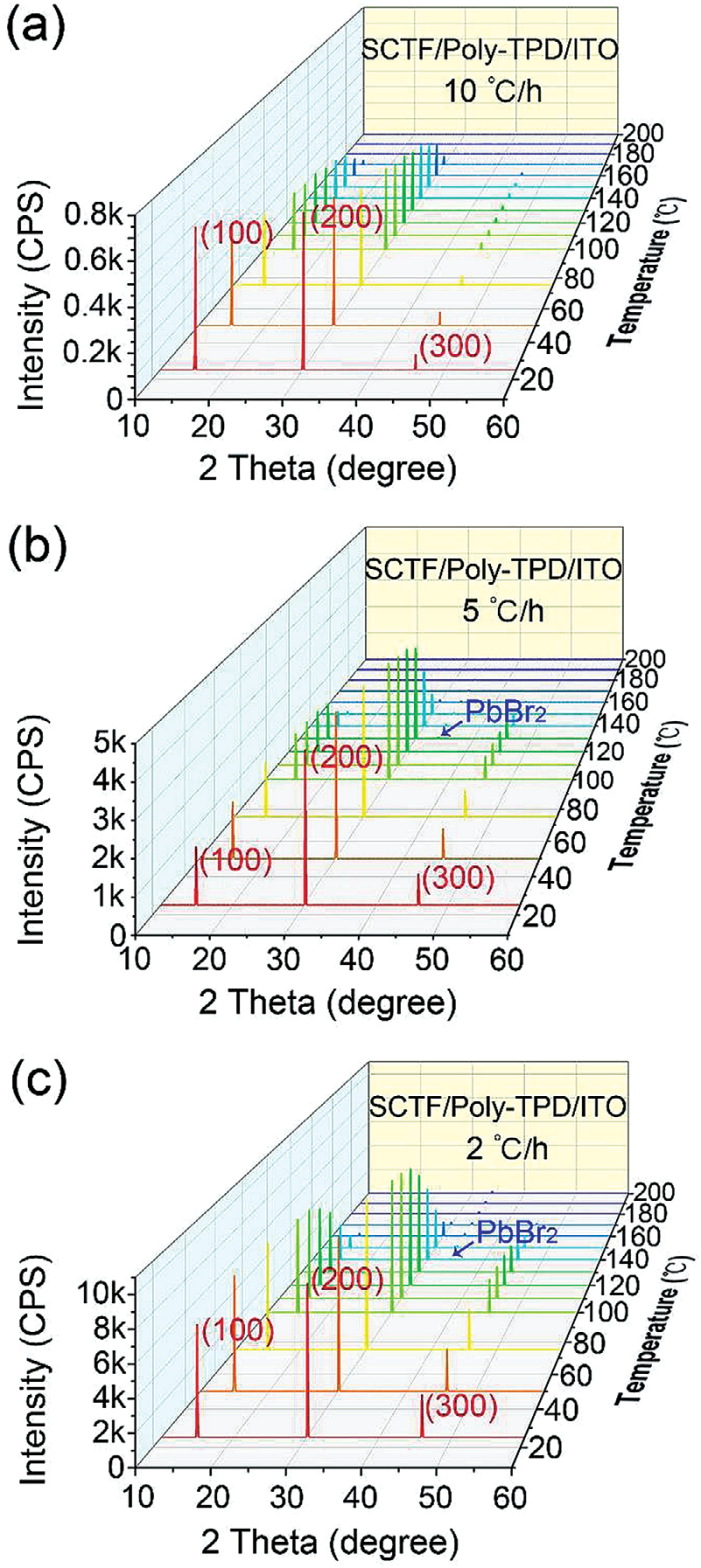
In situ TDXRD patterns of the MAPbBr_3_ SCTFs grown on poly‐TPD/ITO under a dead load of 2 kg at three heating rates of a) 10, b) 5, and c) 2 °C h^−1^, respectively.

The thermal stability of the MAPbBr_3_ SCTFs grown on Poly‐TPD/ITO under a dead load of 2 kg was examined via in‐situ temperature‐dependent XRD (TDXRD) analysis. **Figure** **3** presents in‐situ TDXRD patterns of the MAPbBr_3_ SCTFs grown on poly‐TPD/ITO at the three heating rates (2, 5, and 10 °C h^−1^). All the three MAPbBr_3_ SCTFs exhibit high single crystallinity with three characteristic peaks centered at ≈15.3, ≈30.8, and ≈46.5°, which correspond to the (100), (200), and (300) planes of cubic MAPbBr_3_ crystals. At the same temperature, the intensities of the diffraction peaks decrease with the increase of the heating rate. The peak intensity of the (100) plane for the MAPbBr_3_ SCTFs grown at 2 °C h^−1^ is about three times and nine times larger than that grown at 5 and 10 °C h^−1^, respectively. A similar trend is also observed for (200) and (300) planes. A new PbBr_2_ phase was formed at 140 and 130 °C from the MAPbBr_3_ SCTFs grown at 2 and 5 °C h^−1^, respectively. This result suggests better thermal stability of the MAPbBr_3_ SCTFs grown at 2 °C h^−1^ than that grown at 5 °C h^−1^. Note that there was no PbBr_2_ phase formed from the MAPbBr_3_ SCTFs grown at 10 °C h^−1^ during the TDXRD characterization, which is due to that the inferior crystal grown at 10 °C h^−1^ results in a poor quality of PbBr_2_ crystal, which cannot be detected in the TDXRD, but can be detected in ordinary thin film path as shown Figure S20 in the Supporting Information. From above discussion, it is concluded that smaller heating rates lead to better crystallinity and stability of the MAPbBr_3_ SCTFs.

The optical images of the SCTF morphologies grown with different precursor concentrations under a heating rate of 2 °C h^−1^ and a dead load of 2 kg are shown in Figure S21 (Supporting Information). It is evident that the area of the film on poly‐TPD/ITO increases with the increase of the precursor concentration. The precursor solution with a concentration of 0.2 mol L^−1^ produced the smallest film, while the solution with the concentration of 1.2 mol L^−1^ produced the largest film. According to the nucleation theory, the larger the initial concentration of the precursor solution, the smaller the nucleation barrier and the larger the nucleation rate. At the crystal growth stage, the initial concentration of the precursor is large, leading to a fast crystal growth and a high crystal yield. That is, the size of the SCTFs formed within the same growth duration is larger for a higher initial concentration of the precursor. Such a trend is consistent with the results shown in Figure S21 (Supporting Information) that the size of SCTF gradually increases with the increase of concentration. However, an excessively high initial precursor concentration can cause excessively rapid crystal growth, leading to unstable growth and cracks. For example, the SCTF grown with a precursor concentration of 1.2 mol L^−1^ exhibits ploy‐crystalline feature, as shown in Figure S21d (Supporting Information). According to the crystal growth theory, oversaturated solution (“oversaturation zone” in Figure S22, Supporting Information) will cause a rapid crystal growth. The monomers are in a nonequilibrium state in the crystal, leading to a crystal of poor quality and observable defects such as the grain boundaries. On the other hand, a thermodynamic equilibrium state exists at the “unsaturated zone” with no formation of a solid phase. When a high temperature is introduced, the solution might not be in thermodynamic equilibrium, giving rise to the formation of solid phase with the decrease of solubility, which is referred to as the “metastable zone.” It is preferable for crystal growth in the metastable zone, i.e., the growth of single‐crystalline thin films in Figure S21a–c (Supporting Information) might occur in this region. The corresponding size distribution of SCTFs with various solution concentrations are statistically summarized in Figure S23 (Supporting Information). During the in situ growth of hybrid perovskite SCTFs by the space confinement method, the thickness of the SCTFs is determined by the size of the space (distance between two substrates).^[^ ^17^, ^35^, ^36^, ^37^ ^]^ Therefore, the pressure between the two substrates can be tuned by changing the distance between two substrates. In this way, the thickness of the films was adjusted.^[^ ^17^, ^35^, ^36^, ^37^ ^]^ To explore the influence of low precursor solution concentration on the thickness of the prepared SCTFs, we further measured the thickness of MAPbBr_3_ SCTFs prepared with low precursor solution concentration (0.2 mol L^−1^) were measured to be ≈525 nm, as shown in Figure S24 (Supporting Information), a quite small reduction compared with that of films by larger concentration (0.8 mol L^−1^, Figure S4 in the Supporting Information). The results suggest that the precursor solution concentration almost have no influence on the thickness of SCTFs within a range of 0.2–0.8 mol L^−1^. This is consistent with the previously reports. ^[^ ^17^, ^35^, ^36^, ^37^ ^]^

**Figure 4:**
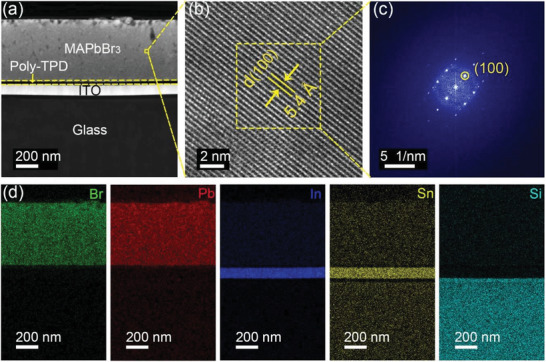
a) STEM image of a MAPbBr_3_ SCTF grown on poly‐TPD/ITO at 2 °C h^−1^ under a dead load of 2 kg, b) HRTEM image of the SCTF of region enclosed by a yellow box in (a), c) SAED pattern of the region enclosed by the yellow box in (b), and d) EDS enclosed mappings of the MAPbBr_3_ SCTF in (a).

From the above results and analysis, a “sweet point”—optimal conditions for the growth of MAPbBr_3_ SCTFs are obtained as 1) the substrates with low interface energy are suggested to be used (such as Poly‐TPD); 2) the concentration of precursor solution should be moderate (≈0.8 mol L^−1^); 3) the heating rate is supposed to be low (2 °C h^−1^). **Figure** **4**a depicts a high‐angle annular dark‐field scanning transmission electron microscopy (HAADF‐STEM) image of the cross‐section of a MAPbBr_3_ SCTF grown on a poly‐TPD/ITO substrate at 2 °C h^−1^ under a dead load of 2 kg. It can be seen that the SCTF is in close contact with the substrate without a clear gap, illustrating the occurrence of heterogeneous nucleation directly on the substrate along with the in situ growth from bottom to top. This image demonstrates the existence of a poly‐TPD layer with a thickness of ≈50 nm, sandwiched between the ITO substrate and the MAPbBr_3_ SCTFs. A high‐resolution transmission electron microscope (HRTEM) image of the region enclosed in the yellow box of Figure 4a is presented in Figure 4b. The HRTEM image shows distinct lattice fringes with a lattice spacing of 5.4 Å, corresponding to the (100) plane of cubic MAPbBr_3_ crystal. The selected area electron diffraction (SAED) pattern of the region enclosed by the yellow box in Figure 4b is shown in Figure 4c. The regularly arranged spots in this SAED pattern indicate MAPbBr_3_ film are single crystalline, with the circled spot corresponding to (100) plane. Both the HRTEM image and the SAED pattern confirm the excellent crystallinity of this MAPbBr_3_ SCTF. The energy‐dispersive X‐ray spectroscopy (EDS) mapping of the cross section image in Figure 4a is shown in Figure 4d. Br and Pb are uniformly distributed in the MAPbBr_3_ SCTF, and In and Sn are uniformly distributed in the ITO substrate. The atomic ratio of Pb to Br is ≈1:3 (Table S6, Supporting Information), confirming the composition of MAPbBr_3_. The presence of In, Sn and Si in the elemental mapping of the MAPbBr_3_ SCTF is likely due to the local decomposition of ITO and soda‐lime glass under the X‐ray irradiation.

**Figure 5:**
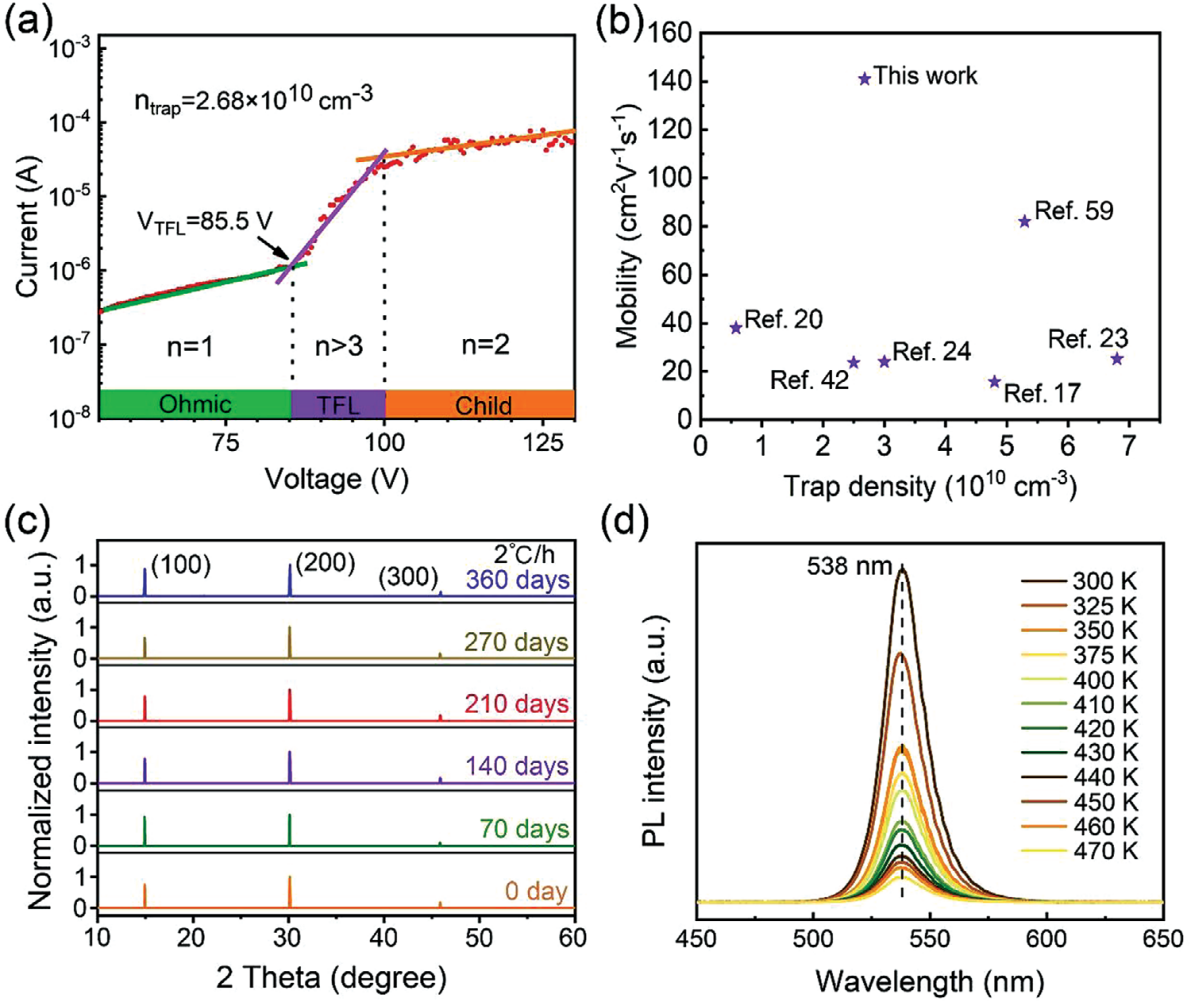
a) *I*–*V* plot of MAPbBr_3_ SCTFs with various regions achieved from the log *I* versus *V*. b) MAPbBr_3_ SCTFs Mobility of this work compared with reported MAPbBr_3_ bulk single crystals or SCTFs. c,d) Stability test of a MAPbBr_3_ SCTF grown on poly‐TPD/ITO at 2°C h^−1^ under a dead load of 2 kg. c) XRD patterns over a period of 360 days, and d) PL thermal stability test through a range of 300 to 470 K.

The trap density of the as‐synthesized MAPbBr_3_ SCTFs was estimated by the dark current–voltage (*I*–*V*) characteristic, with the space charge‐limited current (SCLC) method.^[^ ^23^, ^24^, ^58^ ^]^ As shown in **Figure** **5**a, the *I*−*V* plot, consisting of a linear ohmic response (low voltage), a trap‐filled limit (TFL) region (moderate voltage) and a child region (high voltage) of the MAPbBr_3_ SCTFs is obtained. A remarkable rise of the current in the TFL section indicates a rapid filling of traps.^[^ ^26^ ^]^ From the *V*_TFL_ (85.5 V), the trap density of MAPbBr_3_ SCTFs was estimated to be 2.68 × 10^10^ cm^−3^ (see the Experimental Section for details of calculation) which is comparable to or even lower than the reported results (Figure 5b).^[^ ^17^, ^20^, ^23^, ^24^, ^42^, ^59^ ^]^ On top of this, the carrier mobility of MAPbBr_3_ SCTFs was tested to be 141 cm^2^ V^−1^ s^−1^, which is the largest one achieved so far (Figure 5b).

XRD analyses were further performed to examine the long‐term phase stability of the MAPbBr_3_ SCTFs grown on poly‐TPD/ITO at 2 °C h^−1^ under a dead load of 2 kg over a period of 360 days in an ambient environment (22 °C and 57% RH). As shown in Figure 5c, no observable changes to the diffraction peaks and their intensities over this period is observed. The tested MAPbBr_3_ SCTFs maintained their cubic structure and exhibited excellent long‐term phase stability. Thermal PL stability test was carried out as well. As shown in Figure 5d, the PL peak remains at 538 nm from 300 to 470 K with no shift of the PL peak. This implies that the MAPbBr_3_ SCTFs can maintain a fixed emission‐peak wavelength even at a high temperature of 470 K. It is also observed that the intensity of PL declines with increasing temperature, which is related to exciton dissociation.^[^ ^60^ ^]^ Generally, the decomposition of perovskite crystal thin films is related to their crystalline quality, the concentration of defect states, and the grain boundaries of the crystal. For the same substance, a higher crystal quality results in a lower defect states concentration and fewer grain boundaries, giving rise to a higher decomposition temperature of the crystal.^[^ ^61^, ^62^ ^]^ For example, previous reports pointed out that when MAPbI_3_ polycrystalline films are stored under the ambient conditions, water and oxygen molecules preferentially attack the grain boundaries, and then attack the surface, resulting in complete decomposition of the polycrystalline films.^[^ ^61^, ^62^ ^]^ However, the MAPbBr_3_ SCTFs in the present work have almost no grain boundaries, and it is difficult for water and oxygen molecules to directly attack and decompose these SCTFs. Moreover, previous reports have also mentioned that perovskite films with higher crystalline quality and lower the defect states concentration have higher ion bonding energies, higher decomposition energies and better ambient stability.^[^ ^17^, ^23^, ^35^, ^38^, ^61^, ^62^ ^]^ This explains the excellent long‐term ambient stability of the MAPbBr_3_ SCTFs in this work (Figure 5c). It is worth noting that the thermal stability of MAPbBr_3_ single crystals in humid air was investigated previously upon the local surface degradation through AFM and conductive atomic force microscopy (CAFM).^[^ ^63^ ^]^ The investigation pointed out that large craters with about 5 µm in size was produced on the surface of MAPbBr_3_ single crystals under a temperature of 363 K for 1 h, which corresponds to the CAFM results, in which current level obtained from *I−V* curve was lower than that of a fresh one.^[^ ^63^ ^]^ However, the authors did not characterize the crystalline quality, the concentration of defect states and the number of grain boundaries of their crystals. In our work, we jointly characterized the crystalline quality, trap density and number of grain boundaries of the crystal films by XRD rocking‐curve, SCLC, TRPL, and SEM, which prove that our MAPbBr_3_ single crystal films have high crystallinity (0.017° for FWHM of XRD peak and 1552 ns for average carrier lifetime) and low trap density (2.68 × 10^10^ cm^−3^) and almost no grain boundaries. As a result, the synthesized MAPbBr_3_ single crystal films in the present work exhibit a high environmental stability.

## 3 Conclusion

In conclusion, in situ growth of high‐quality and high‐ATT ratio MAPbBr_3_ SCTFs on HTLs are successfully realized through finding the “sweet spot” for crystal growth by exploiting the three key factors of growth temperature, precursor solution concentration, and the interface energy between the two phases. The results present that high‐quality large‐size SCTFs favor small interface energy between precursor solution and substrate, low heating rate and moderate precursor solution concentration. Besides, the DFT calculations reveal the hydrogen bonds interaction between MAPbBr_3_ SCTFs and HTLs, suggesting the feasibility of growing MAPbBr_3_ SCTFs over different conducting polymers via the hydrogen bonds epitaxial growth. Sub‐centimeter‐scale MAPbBr_3_ SCTFs growth on poly‐TPD HTLs is realized by taking advantage of the influence of these key factors. The MAPbBr_3_ SCTFs grown on poly‐TPD at a small heating rate of 2 °C h^−1^ exhibit desirable morphology with a recorded ratio of ATT of 1.94 × 10^4^ mm (thickness is 540 nm) and outstanding thermal and long‐term structure stabilities and excellent crystallinity with a ultralong average carrier lifetime of 1552 ns ever reported, which benefits from excellent crystallinity with FWHM of 0.017° of XRD peak. The small heating rate allowed ions in the precursor solution to have enough time to reach equilibrium sites during the growth of the films. The microstructure of the MAPbBr_3_ SCTFs remained almost the same after 360 days in ambient environment, during which there were no phase transformation and structural degradation. The results from the thermal PL stability test indicates that the MAPbBr_3_ SCTFs can remain a good pure emission color at a high temperature of 470 K. This work provides an effective and constructive crystallization method for optoelectronic devices fabrication based on perovskite SCTFs.

## 4 Experimental Section

### Preparation of Perovskite Precursor Solution

Equimolar CH_3_NH_3_Br (99.5%, Aladdin) and PbBr_2_ (99.0%, Aladdin) were dissolved in dimethylformamide (DMF, >99% Shanghai Ling Feng Chemical Reagent Co., Ltd.) at 25 °C to obtain a 0.8 mol L^−1^ solution. After vigorous stirring for 2 h, a clear solution was obtained. Precursor solutions with other concentrations were prepared by the same method.

### Preparation of Supersaturated Perovskite Precursor Solution

Equimolar CH_3_NH_3_Br (99.5%, Aladdin) and PbBr_2_ (99.0%, Aladdin) were dissolved in dimethyl formamide (DMF, >99% Shanghai Ling Feng Chemical Reagent Co., Ltd.) at 60 °C to obtain a 1.6 mol L^−1^ supersaturated solution. After vigorous stirring 16 h, a clear solution was obtained.

### Preparation of MAPbBr_3_ SCTFs

The MAPbBr_3_ SCTFs were prepared by multiple steps, as schematically illustrated in Figure S25 (Supporting Information): 1) spin‐coating a HTL (poly‐TPD, TFB or PEDOT:PSS) on an ITO glass (3000 rpm for 45 s) at room temperature; 2) annealing the HTL/ITO sample at 130 °C for 10 min in glove box with nitrogen gas to evaporate the solvent (chlorobenzene) and enhance the homogeneity; 3) sandwiching of the MAPbBr_3_ precursor solution of 60 µL between two identical HTL‐coated ITO glass slides of 32 × 26 mm^2^ with the HTLs being in direct contact with the precursor solution under a dead load of 2 kg; 4) heating the sandwiched structure to 90 °C at a heating rate of 10, 5, or 2 °C h^−1^ and maintaining temperature at 90 °C for 2 h; and 5) annealing the sandwiched structure at 60 °C for 12 h. Then, the films were obtained by separating the two HTL‐coated ITO glass substrates by hand gently for further characterizations. ^[^ ^17^, ^35^, ^36^, ^37^ ^]^ In this separation process, since the precursor solution has been completely consumed, the interaction between the film and the substrate is relatively weak, mainly in the form of hydrogen bonds (Figure S9, Supporting Information). There is still a quite small gap between the two glass slides, the perovskite films can be achieved intactly.^[^ ^17^, ^35^, ^36^, ^37^ ^]^

### Characterizations

The thickness characterization of the formed MAPbBr_3_ SCTFs was conducted on a step profiler (Dektalk XT, Bruker). The morphologies of the MAPbBr_3_ SCTFs were characterized on a field‐emission scanning electron microscope (FESEM) (Gemini SEM 300). The structure of the MAPbBr_3_ SCTFs was analyzed on a high‐resolution transmission electron microscope (HRTEM) (FEI, Tecnai F30) equipped with a select area electron diffraction‐meter (SAED). The surface roughness of the MAPbBr_3_ SCTFs was measured on an atomic force microscope (AFM) (MFP‐3D‐Stand Alone, Asylum Research). The XRD and TDXRD measurements of the MAPbBr_3_ SCTFs were conducted on an X‐ray diffractometer (Rigaku Smartlab) with Cu K_*α*1_ radiation (*λ* = 1.54056 Å), *K*_*α*2_ radiation (*λ* = 1.54439 Å) (average *λ* = 1.5418 Å). The time‐resolved photoluminescence (TRPL) measurement was conducted on a fluorescence lifetime spectrometer (PicoQuant) with a laser of 405 nm in wavelength. Surface tension and contact angles were measured on a surface tension meter (JK99B, Powereach) and contact angle meter (JC200C1, Powereach), respectively. Carrier mobility was tested by a Hall effect measurement system (Nanometric HL5500PC).

### *I*–*V* Measurement and Trap Density Calculation

The *I*–*V* test for the trap density calculation was carried out at room temperature in a dark environment by a parameter analyzer (4200A‐SCS, Keithley) on a SCTF (3 mm in thickness) sandwiched by Au electrodes (150 nm in thickness). The density of trap (*n*_trap_) is calculated by^[^ ^24^, ^58^ ^]^

(18) $$n_{trap}=2\varepsilon \varepsilon_{0}V_{TFL}/qL^{2}$$

where *ε* is the dielectric (25.5),^[^ ^23^, ^24^, ^64^ ^]^ *ε*_0_ is the vacuum permittivity, q is the electronic charge, L is the thickness of the crystal film.

### Density Functional Theory Calculation

Using the spin‐polarization density functional theory (DFT)^[^ ^65^, ^66^ ^]^ with the Perdew–Burke–Ernzerhof (PBE) formulation,^[^ ^67^ ^]^ the first‐principle calculation was performed to analyze the interaction between a conducting polymer (hole‐transport layer) and MAPbBr_3_ SCTFs within generalized gradient approximation. The projected augmented wave (PAW) potentials^[^ ^68^, ^69^ ^]^ was used to describe the ionic cores and took valence electrons into account using a plane wave basis set with a kinetic energy cutoff of 400 eV. Partial occupancies of the Kohn−Sham orbitals were allowed via the Gaussian smearing method and a width of 0.05 eV. The electronic energy was considered as self‐consistent for the energy change smaller than 10^−6^ eV. A geometric optimization was considered to be convergent for the energy change smaller than 0.05 eV Å^−1^. Five layers of MAPbBr_3_ of (001) plane were used in the calculation to establish the interface of molecule/MAPbBr_3_. The sixth layer was fixed during relaxation. The binding energies (*E*_b_) were calculated as *E*_b_ = *E*_ad/sub_ − *E*_ad_ – *E*_sub_, where *E*_ad/sub_, *E*_ad_, and *E*_sub_ are the total energy of the optimized molecule/MAPbBr_3_ interface system and surface energies of the molecule and MAPbBr_3_, respectively.

## Conflict of Interest

The authors declare no conflict of interest.

## Supporting information

Supporting Information

Supplemental Movie 1

## Data Availability

The data that support the findings of this study are available from the corresponding author upon reasonable request.

## References

[1] G. E. Eperon , V. M. Burlakov , P. Docampo , A. Goriely , H. J. Snaith , Adv. Funct. Mater. 2014, 24, 151.

[2] F. Zhang , H. Lu , J. Tong , J. J. Berry , M. C. Beard , K. Zhu , Energy Environ. Sci. 2020, 13, 1154.

[3] M. Jeong , I. W. Choi , E. M. Go , Y. Cho , M. Kim , B. Lee , S. Jeong , Y. Jo , H. W. Choi , J. Lee , J. H. Bae , S. K. Kwak , D. S. Kim , C. Yang , Science 2020, 369, 1615. 32973026 10.1126/science.abb7167

[4] Y. Cao , N. Wang , H. Tian , J. Guo , Y. Wei , H. Chen , Y. Miao , W. Zou , K. Pan , Y. He , H. Cao , Y. Ke , M. Xu , Y. Wang , M. Yang , Z. Fu , D. Kong , D. Dai , Y. Jin , G. Li , H. Li , Q. Peng , J. Wang , W. Huang , Nature 2018, 562, 249. 30305742 10.1038/s41586-018-0576-2

[5] K. Lin , J. Xing , L. Quan , F. P. G. de Arquer , X. Gong , J. Lu , L. Xie , W. Zhao , D. Zhang , C. Yan , W. Li , X. Liu , Y. Lu , J. Kirman , E. H. Sargent , Q. Xiong , Z. Wei , Nature 2018, 562, 245. 30305741 10.1038/s41586-018-0575-3

[6] W. Wei , Y. Zhang , Q. Xu , H. Wei , Y. Fang , Q. Wang , Y. Deng , T. Li , A. Gruverman , L. Cao , J. Song , Nat. Photonics 2017, 11, 315.

[7] G. Xing , N. Mathews , S. S. Lim , N. Yantara , X. Liu , D. Sabba , M. Grätzel , S. Mhaisalkar , T. C. Sum , Nat. Mater. 2014, 13, 476. 24633346 10.1038/nmat3911

[8] D. W. de Quilettes , S. M. Vorpahl , S. D. Stranks , H. Nagaoka , G. E. Eperon , M. E. Ziffer , H. J. Snaith , D. S. Ginger , Science 2015, 348, 683. 25931446 10.1126/science.aaa5333

[9] N. J. Jeon , J. H. Noh , W. S. Yang , Y. C. Kim , S. Ryu , J. Seo , S. I. Seok , Nature 2015, 517, 476. 25561177 10.1038/nature14133

[10] F. Ye , H. Chen , F. Xie , W. Tang , M. Yin , J. He , E. Bi , Y. Wang , X. Yang , L. Han , Energy Environ. Sci. 2016, 9, 2295.

[11] T. M. Brenner , D. A. Egger , L. Kronik , G. Hodes , D. Cahen , Nat. Rev. Mater. 2016, 1, 15007.

[12] H. Min , D. Y. Lee , J. Kim , G. Kim , K. S. Lee , J. Kim , M. J. Paik , Y. K. Kim , K. S. Kim , M. G. Kim , T. J. Shin , S. Seok , Nature 2021, 598, 444. 34671136 10.1038/s41586-021-03964-8

[13] A. Al‐Ashouri , E. Kohnen , B. Li , A. Magomedov , H. Hempel , P. Caprioglio , J. A. Marquez , A. B. M. Vilches , E. Kasparavicius , J. A. Smith , N. Phung , D. Menzel , M. Grischek , L. Kegelmann , D. Skroblin , C. Gollwitzer , T. Malinauskas , M. Jost , G. Matic , B. Rech , R. Schlatmann , M. Topic , L. Korte , A. Abate , B. Stannowski , D. Neher , M. Stolterfoht , T. Unold , V. Getautis , S. Albrecht , Science 2020, 370, 1300. 33303611 10.1126/science.abd4016

[14] W. Xu , Q. Hu , S. Bai , C. Bao , Y. Miao , Z. Yuan , T. Borzda , A. J. Barker , E. Tyukalova , Z. Hu , M. Kawecki , H. Wang , Z. Yan , X. Liu , X. Shi , K. Uvdal , M. Fahlman , W. Zhang , M. Duchamp , J. Liu , A. Petrozza , J. Wang , L. Liu , W. Huang , F. Gao , Nat. Photonics 2019, 13, 418.

[15] Z. Xiao , Y. Yuan , Y. Shao , Q. Wang , Q. Dong , C. Bi , P. Sharma , A. Gruverman , J. Huang , Nat. Mater. 2015, 14, 193. 25485985 10.1038/nmat4150

[16] C. Eames , J. M. Frost , P. R. Barnes , B. C. O'regan , A. Walsh , M. S. Islam , Nat. Commun. 2015, 6, 7497. 26105623 10.1038/ncomms8497 PMC4491179

[17] Y. Chen , Q. Ge , Y. Shi , J. Liu , D. Xue , J. Ma , J. Ding , H. Yan , J. Hu , L. Wan , J. Am. Chem. Soc. 2016, 138, 16196. 27998083 10.1021/jacs.6b09388

[18] Q. Dong , Y. Fang , Y. Shao , P. Mulligan , J. Qiu , L. Cao , J. Huang , Science 2015, 347, 967. 25636799 10.1126/science.aaa5760

[19] Z. Guo , Y. Wan , M. Yang , J. Snaider , K. Zhu , L. Huang , Science 2017, 356, 59. 28386007 10.1126/science.aam7744

[20] D. Shi , V. Adinolfi , R. Comin , M. Yuan , E. Alarousu , A. Buin , Y. Chen , S. Hoogland , A. Rothenberger , K. Katsiev , Y. Losovyj , X. Zhang , P. A. Dowben , O. F. Mohammed , E. H. Sargent , O. M. Bakr , Science 2015, 347, 519. 25635092 10.1126/science.aaa2725

[21] Z. Lian , Q. Yan , T. Gao , J. Ding , Q. Lv , C. Ning , Q. Li , J. Sun , J. Am. Chem. Soc. 2016, 138, 9409. 27458057 10.1021/jacs.6b05683

[22] Q. Han , S. H. Bae , P. Sun , Y. T. Hsieh , Y. Yang , Y. S. Rim , H. Zhao , Q. Chen , W. Shi , G. Li , Y. Yang , Adv. Mater. 2016, 28, 2253. 26790006 10.1002/adma.201505002

[23] Y. Liu , Y. Zhang , Z. Yang , J. Feng , Z. Xu , Q. Li , M. Hu , H. Ye , X. Zhang , M. Liu , K. Zhao , S. Liu , Mater. Today 2019, 22, 67.

[24] M. I. Saidaminov , A. L. Abdelhady , B. Murali , E. Alarousu , V. M. Burlakov , W. Peng , I. Dursun , L. Wang , Y. He , G. Maculan , A. Goriely , T. Wu , O. F. Mohammed , O. M. Bakr , Nat. Commun. 2015, 6, 7586. 26145157 10.1038/ncomms8586 PMC4544059

[25] W. Peng , L. Wang , B. Murali , K. T. Ho , A. Bera , N. Cho , C. F. Kang , V. M. Burlakov , J. Pan , L. Sinatra , C. Ma , W. Xu , D. Shi , E. Alarousu , A. Goriely , J. He , O. F. Mohammed , T. Wu , O. M. Bakr , Adv. Mater. 2016, 28, 3383. 26931100 10.1002/adma.201506292

[26] Z. Gu , Z. Huang , C. Li , M. Li , Y. Song , Sci. Adv. 2018, 4, eaat2390. 29963635 10.1126/sciadv.aat2390 PMC6025903

[27] X. Xiao , J. Dai , Y. Fang , J. Zhao , X. Zheng , S. Tang , P. N. Rudd , X. C. Zeng , J. Huang , ACS Energy Lett. 2018, 3, 684.

[28] Z. Yang , Y. Deng , X. Zhang , S. Wang , H. Chen , S. Yang , J. Khurgin , N. X. Fang , X. Zhang , R. Ma , Adv. Mater. 2018, 30, 1704333. 10.1002/adma.201704333 29315842

[29] M. V. Kelso , N. K. Mahenderkar , Q. Chen , J. Z. Tubbesing , J. A. Switzer , Science 2019, 364, 166. 30975885 10.1126/science.aaw6184

[30] A. A. Zhumekenov , V. M. Burlakov , M. I. Saidaminov , A. Alofi , M. A. Haque , B. Turedi , B. Davaasuren , I. Dursun , N. Cho , A. M. El‐Zohry , M. De Bastiani , A. Giugni , B. Torre , E. Di Fabrizio , O. F. Mohammed , A. Rothenberger , T. Wu , A. Goriely , O. M. Bakr , ACS Energy Lett. 2017, 2, 1782.

[31] K. Wang , C. Wu , D. Yang , Y. Jiang , S. Priya , ACS Nano 2018, 12, 4919. 29683643 10.1021/acsnano.8b01999

[32] Y. Liu , Q. Dong , Y. Fang , Y. Lin , Y. Deng , J. Huang , Adv. Funct. Mater. 2019, 29, 1807707.

[33] O. Malinkiewicz , A. Yella , Y. H. Lee , G. M. Espallargas , M. Graetzel , M. K. Nazeeruddin , H. J. Bolink , Nat. Photonics 2014, 8, 128.

[34] R. Po , C. Carbonera , A. Bernardi , N. Camaioni , Energy Environ. Sci. 2011, 4, 285.

[35] Z. Chen , Q. Dong , Y. Liu , C. Bao , Y. Fang , Y. Lin , S. Tang , Q. Wang , X. Xiao , Y. Bai , Y. Deng , J. Huang , Nat. Commun. 2017, 8, 1890. 29192232 10.1038/s41467-017-02039-5 PMC5709415

[36] A. Y. Alsalloum , B. Turedi , X. Zheng , S. Mitra , A. A. Zhumekenov , K. J. Lee , P. Maity , I. Gereige , A. AlSaggaf , I. S. Roqan , O. F. Mohammed , O. M. Bakr , ACS Energy Lett. 2020, 5, 657.

[37] A. Y. Alsalloum , B. Turedi , K. Almasabi , X. Zheng , R. Naphade , S. D. Stranks , O. F. Mohammed , O. M. Bakr , Energy Environ. Sci. 2021, 14, 2263.

[38] Z. Chen , B. Turedi , A. Y. Alsalloum , C. Yang , X. Zheng , I. Gereige , A. AlSaggaf , O. F. Mohammed , O. M. Bakr , ACS Energy Lett. 2019, 4, 1258.

[39] S. D. Stranks , G. E. Eperon , G. Grancini , C. Menelaou , M. J. Alcocer , T. Leijtens , L. M. Herz , A. Petrozza , H. J. Snaith , Science 2013, 342, 341. 24136964 10.1126/science.1243982

[40] P. Docampo , J. M. Ball , M. Darwich , G. E. Eperon , H. J. Snaith , Nat. Commun. 2013, 4, 2761. 24217714 10.1038/ncomms3761

[41] X. Jiang , X. Fu , D. Ju , S. Yang , Z. Chen , X. Tao , ACS Energy Lett. 2020, 5, 1797.

[42] H. Rao , B. Chen , X. Wang , D. Kuang , C. Su , Chem. Commun. 2017, 53, 5163. 10.1039/c7cc02447a 28439587

[43] Z. Xiao , D. Wang , Q. Dong , Q. Wang , W. Wei , J. Dai , X. Zeng , J. Huang , Energy Environ. Sci. 2016, 9, 867.

[44] N. J. Jeon , J. H. Noh , Y. C. Kim , W. S. Yang , S. Ryu , S. I. Seok , Nat. Mater. 2014, 13, 897. 24997740 10.1038/nmat4014

[45] I. V. Markov , Crystal Growth for Beginners: Fundamentals of Nucleation, Crystal Growth and Epitaxy, World Scientific, Singapore 2016.

[46] M. H. Sung , J. S. Kim , W. S. Kim , I. Hirasawa , W. S. Kim , J. Cryst. Growth 2002, 235, 529.

[47] D. K. Owens , R. Wendt , J. Appl. Polym. Sci. 1969, 13, 1741.

[48] T. Young , Philos. Trans. R. Soc. London 1805, 95, 65.

[49] F. Yang , Phys. Chem. Chem. Phys. 2020, 22, 9990. 32364192 10.1039/d0cp00559b

[50] X. Zhan , M. Shirpour , F. Yang , Electrochim. Acta 2015, 173, 736.

[51] X. Huang , T. R. Paudel , P. A. Dowben , S. Dong , E. Y. Tsymbal , Phys. Rev. B 2016, 94, 195309.

[52] Y. Liu , Z. Yang , S. Liu , Adv. Sci. 2018, 5, 1700471. 10.1002/advs.201700471 PMC5770672 29375973

[53] J. Li , Z. Han , Y. Gu , D. Yu , J. Liu , D. Hu , X. Xu , H. Zeng , Adv. Funct. Mater. 2021, 31, 2008684

[54] Z. Wu , M. Jiang , Z. Liu , A. Jamshaid , L. K. Ono , Y. Qi , Adv. Energy Mater. 2020, 10, 1903696.

[55] H. Fan , F. Li , P. Wang , Z. Gu , J.‐H. Huang , K.‐J. Jiang , B. Guan , L.‐M. Yang , X. Zhou , Y. Song , Nat. Commun. 2020, 11, 5402. 33159051 10.1038/s41467-020-19199-6 PMC7648077

[56] X. Cheng , S. Yang , B. Cao , X. Tao , Z. Chen , Adv. Funct. Mater. 2020, 30, 1905021.

[57] Y. Wu , F. Xie , H. Chen , X. Yang , H. Su , M. Cai , Z. Zhou , T. Noda , L. Han , Adv. Mater. 2017, 29, 1701073. 10.1002/adma.201701073 28524262

[58] R. H. Bube , J. Appl. Phys. 1962, 33, 1733.

[59] Y. Lei , Y. Chen , Y. Gu , C. Wang , Z. Huang , H. Qian , J. Nie , G. Hollett , W. Choi , Y. Yu , N. Kim , C. Wang , T. Zhang , H. Hu , Y. Zhang , X. Li , Y. Li , W. Shi , Z. Liu , M. J. Sailor , L. Dong , Y. H. Lo , J. Luo , S. Xu , Adv. Mater. 2018, 30, 1705992. 10.1002/adma.201705992 29611280

[60] D. Lubyshev , P. González‐Borrero , E. Marega Jr , E. Petitprez , N. La Scala Jr , P. Basmaji , Appl. Phys. Lett. 1996, 68, 205.

[61] Q. Wang , B. Chen , Y. Liu , Y. Deng , Y. Bai , Q. Dong , J. Huang , Energy Environ. Sci. 2017, 10, 516.

[62] A. Mei , Y. Sheng , Y. Ming , Y. Hu , Y. Rong , W. Zhang , S. Luo , G. Na , C. Tian , X. Hou , Y. Xiong , Z. Zhang , S. Liu , S. Uchida , T.‐W. Kim , Y. Yuan , L. Zhang , Y. Zhou , H. Han , Joule 2020, 4, 2646.

[63] G. Mannino , I. Deretzis , E. Smecca , F. Giannazzo , S. Valastro , G. Fisicaro , A. La Magna , D. Ceratti , A. Alberti , J. Phys. Chem. C 2021, 125, 4938.

[64] A. Poglitsch , D. Weber , J. Chem. Phys. 1987, 87, 6373.

[65] G. Kresse , J. Furthmüller , Comput. Mater. Sci. 1996, 6, 15.

[66] G. Kresse , J. Furthmüller , Phys. Rev. B 1996, 54, 11169. 10.1103/physrevb.54.11169 9984901

[67] J. P. Perdew , K. Burke , M. Ernzerhof , Phys. Rev. Lett. 1996, 77, 3865. 10062328 10.1103/PhysRevLett.77.3865

[68] G. Kresse , D. Joubert , Phys. Rev. B 1999, 59, 1758.

[69] P. E. Blöchl , Phys. Rev. B 1994, 50, 17953. 10.1103/physrevb.50.17953 9976227

