# Supplementary material for: In Situ Growth Mechanism for High‐Quality Hybrid Perovskite Single‐Crystal Thin Films with High Area to Thickness Ratio: Looking for the Sweet Spot
Source: Adv Sci (Weinh). 2022 Mar 8;9(13):2104788. doi: 10.1002/advs.202104788 (PMC9069385; doi:10.1002/advs.202104788)
Supplement: Supplementary file 1 — Supporting Information [file ADVS-9-2104788-s001.pdf]

Supporting Information

In Situ Growth Mechanism for High-Quality Hybrid Perovskite Single-Crystal Thin Films with High Area to Thickness Ratio: Looking for the Sweet Spot

Xiaobing Tang,[†] Zhaojin Wang,[†] Dan Wu, Zhenghui Wu, Zhenwei Ren, Ruxue Li, Pai Liu, Guanding Mei, Jiayun Sun, Jiahao Yu, Fankai Zheng, Wallace C. H. Choy, Rui Chen, Xiao Wei Sun, Fuqian Yang,* and Kai Wang**

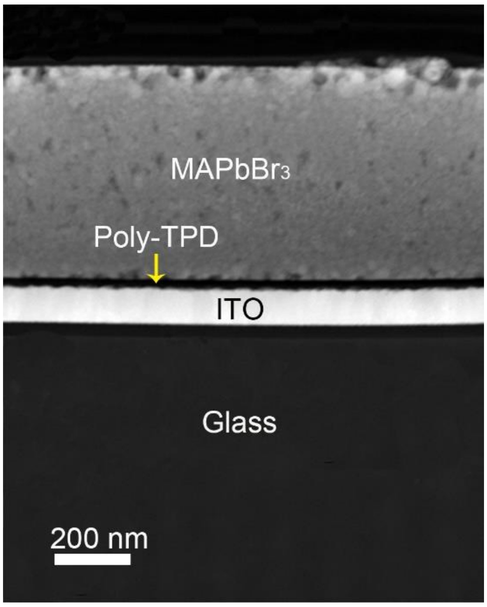

Figure S1. STEM image of a layer of $MAPbBr_3$ SCTF grown on Poly-TPD/ITO substrate.

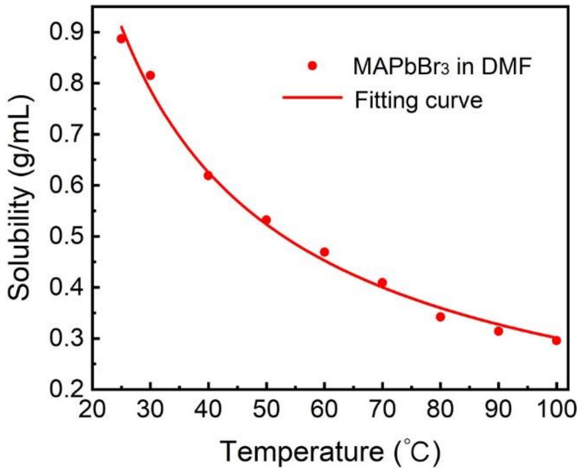

Figure S2. Temperature dependence of the solubility of $MAPbBr_3$ in DMF.

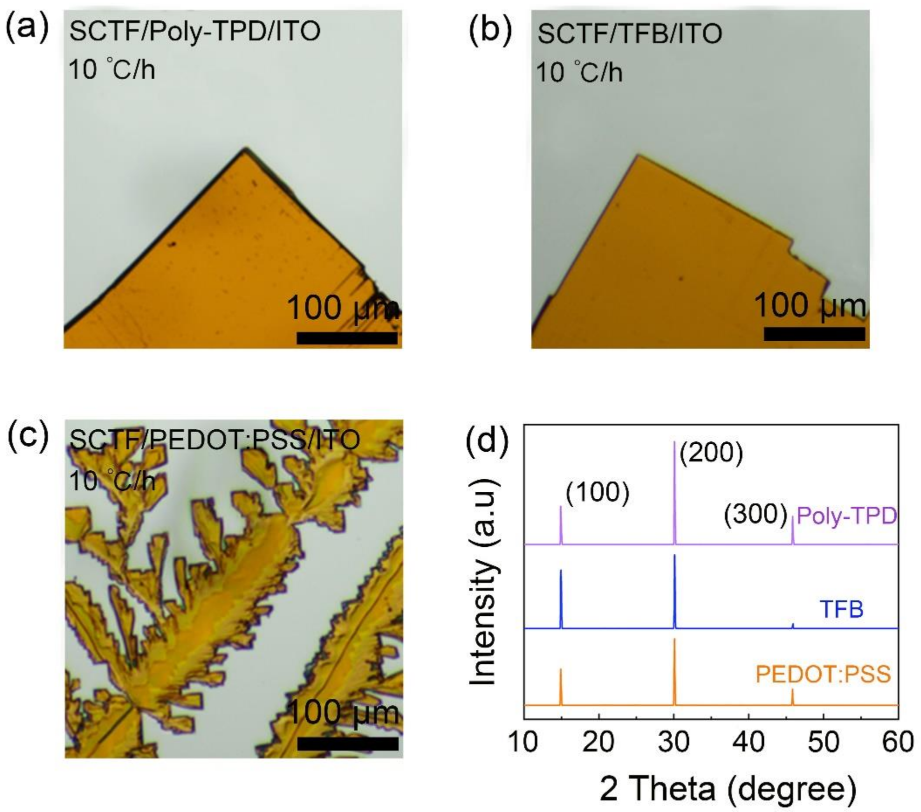

Figure S3. Optical morphologies (a-c) and XRD patterns (d) of the $MAPbBr_3$ SCTFs grown on (a) Poly-TPD, (b) TFB/ITO, and (c) PEDOT:PSS substrates at a heating rate of 10 °C/h.

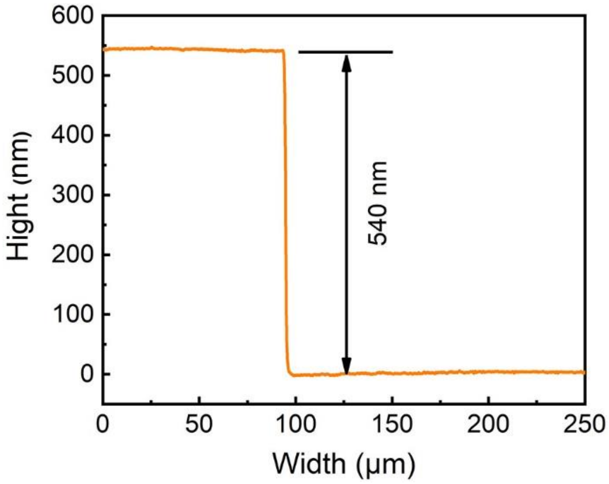

Figure S4. Surface profile of a $MAPbBr_3$ SCTF on Poly-TPD/ITO substrate.

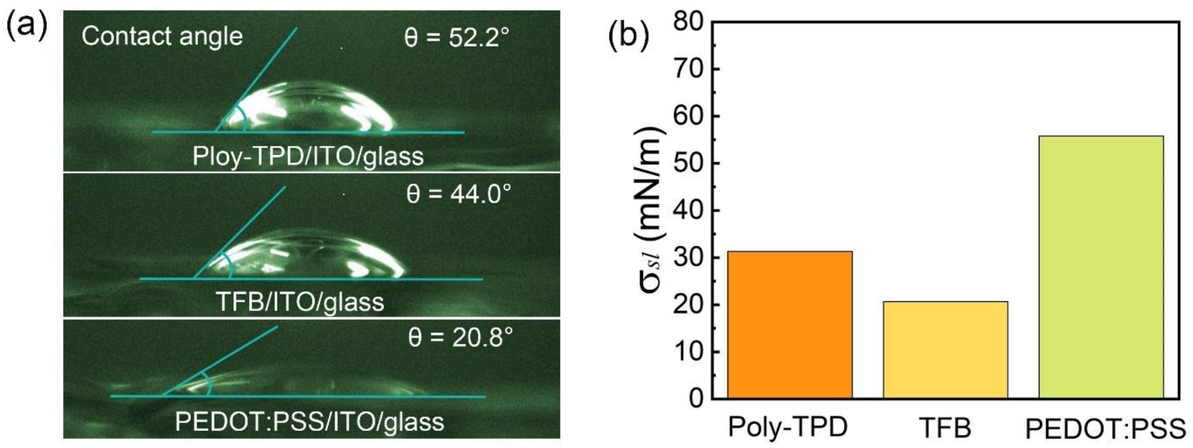

Figure S5. (a) Optic images for the measurement of the contact angles of the precursor solution on three different HTLs, and (b) interface energies between the precursor solution and three different HTLs.

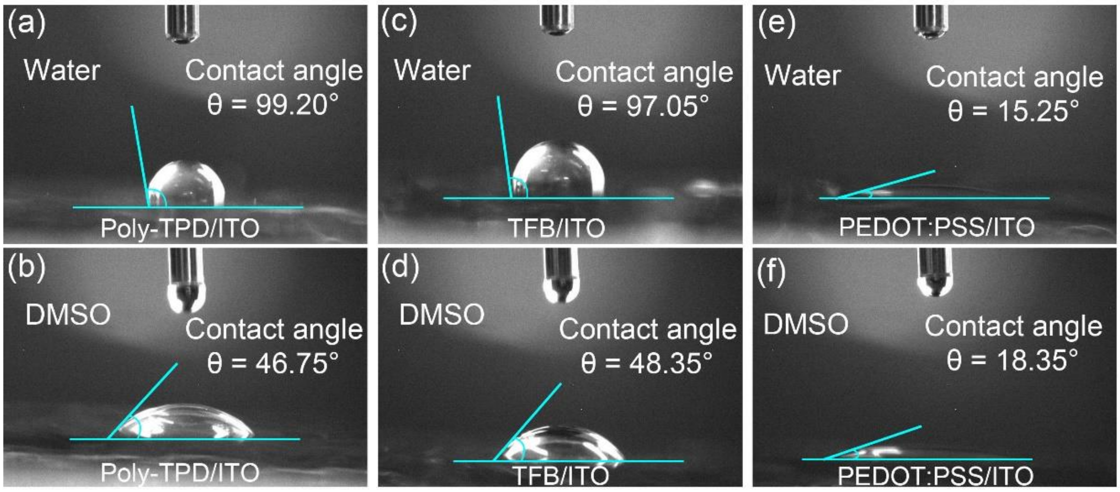

Figure S6. Optical images of contact angles of water and DMSO on various substrates.

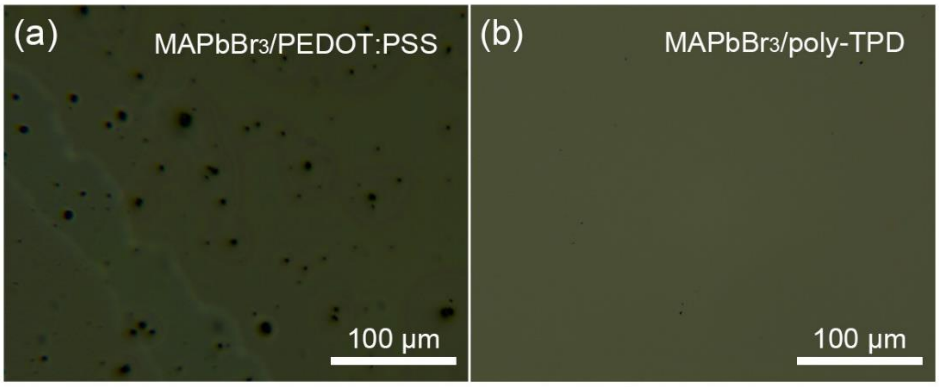

Figure S7. Optical images showing morphologies of $MAPbBr_3$ islands on (a) PEDOT:PSS and uniform $MAPbBr_3$ (b) Poly-TPD substrates respectively where both samples were grown for 15 h.

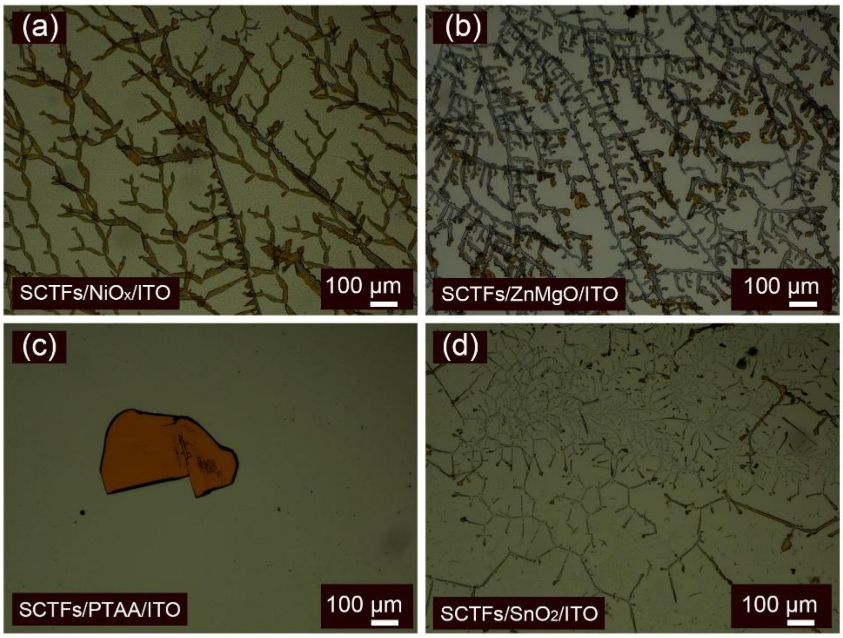

Figure S8. Optical images showing morphologies of $MAPbBr_3$ SCTFs grown on (a) NiOx/ITO glass, (b)ZnMgO/ITO glass, (c) PTAA/ITO glass (d) SnO_2/ITO glass.

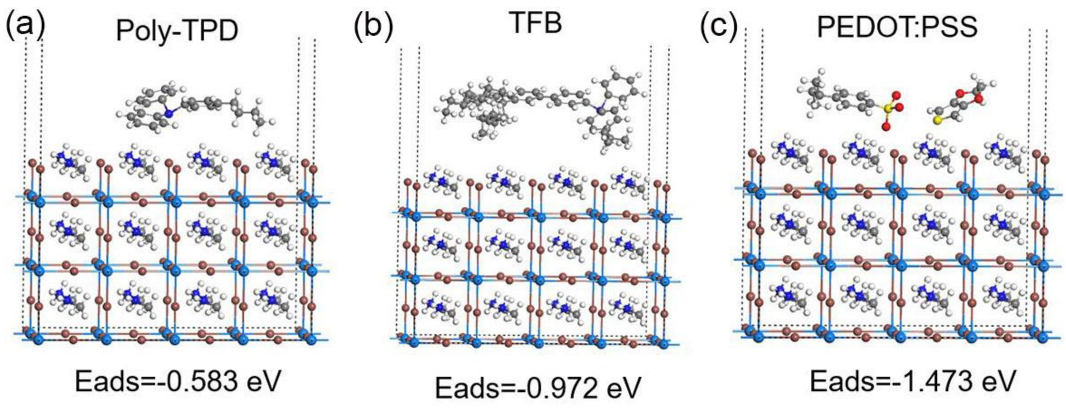

Figure S9. DFT calculations on the interaction between (a) Poly-TPD, (b) TFB, (c) PEDOT:PSS and MABr-terminated $MAPbBr_3$ surfaces (bottom). The corresponding adsorption energy (E_{ads}) for $MAPbBr_3$/Poly-TPD, $MAPbBr_3$/TFB and $MAPbBr_3$/PEDOT:PSS are -0.583 eV (-13.4 kcal/mol), -0.972 eV (-22.4 kcal/mol) and -1.473 eV (-34.0 kcal/mol), respectively.

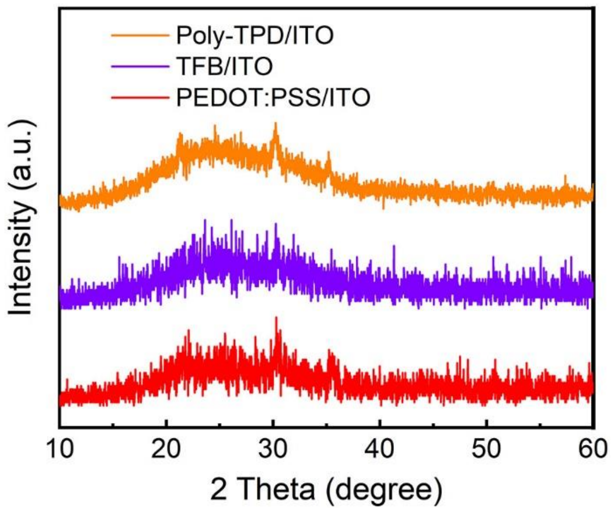

Figure S10. XRD pattern for various substrates.

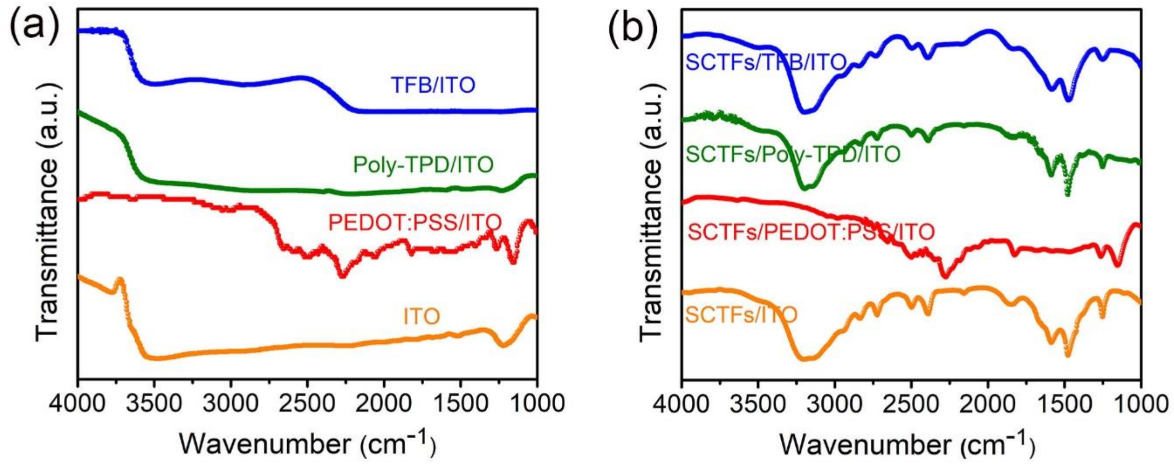

Figure S11. FTIR pattern of various substrates (a) before and (b) after the growth of SCTFs.

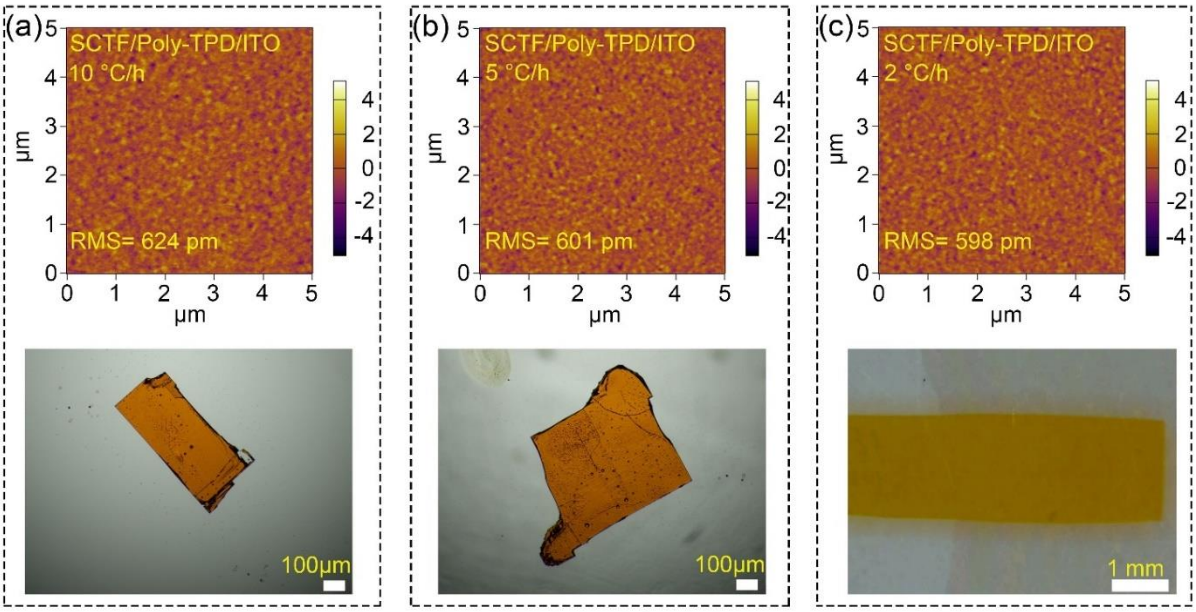

Figure S12. $MAPbBr_3$ SCTFs grown on Poly-TPD/ITO at (a) 10, (b) 5, and (c) 2 °C/h under a dead load of 2 kg. Top: RMS value of various films obtained from an AFM. Bottom: optical images from a microscope.

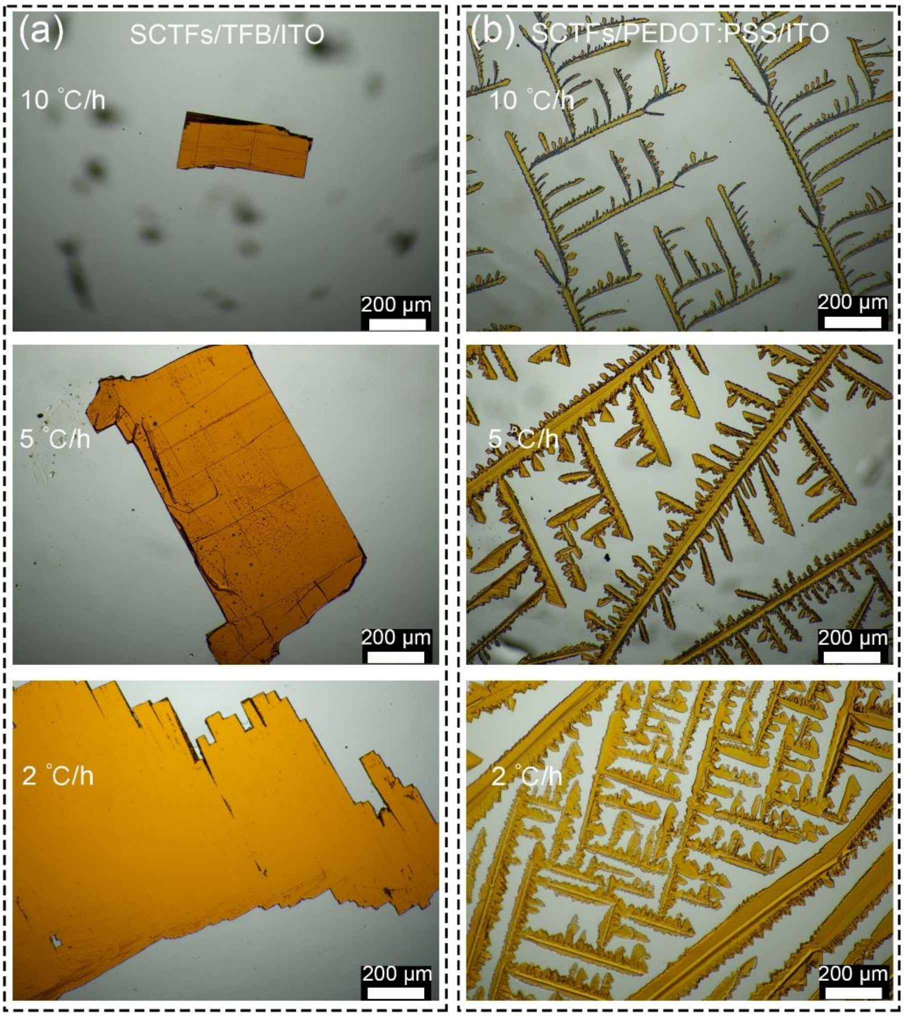

Figure S13. Optical images of $MAPbBr_3$ SCTFs grown on (a) TFB and (b) PEDOT:PSS at heating rates of 10, 5 and 2 ℃/h.

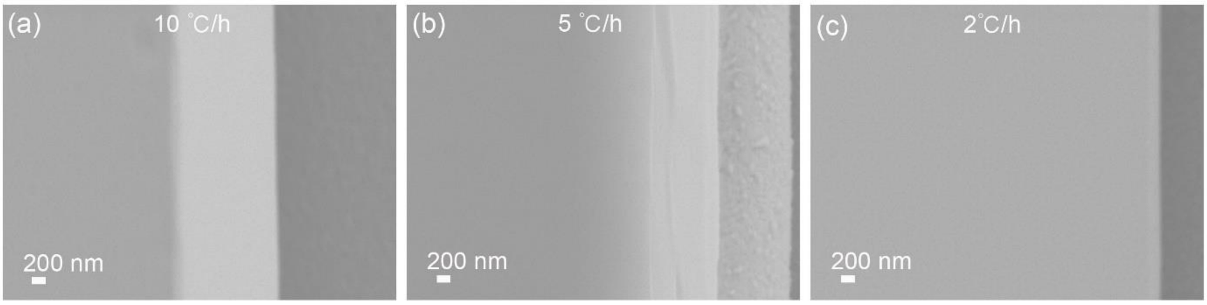

Figure S14. Top-view SEM images of single $MAPbBr_3$ SCTF grown on Poly-TPD/ITO at (a) 10, (b) 5, and (c) 2 °C/h under a dead load of 2 kg. The dark area at right side represents Poly-TPD layer.

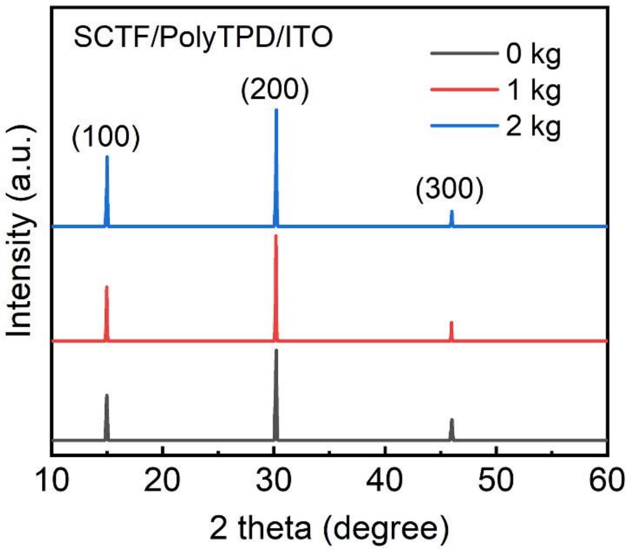

Figure S15. XRD patterns of $MAPbBr_3$ SCTFs prepared under a dead load of 0 kg (black line), 1 kg (red line), and 2 kg (blue line).

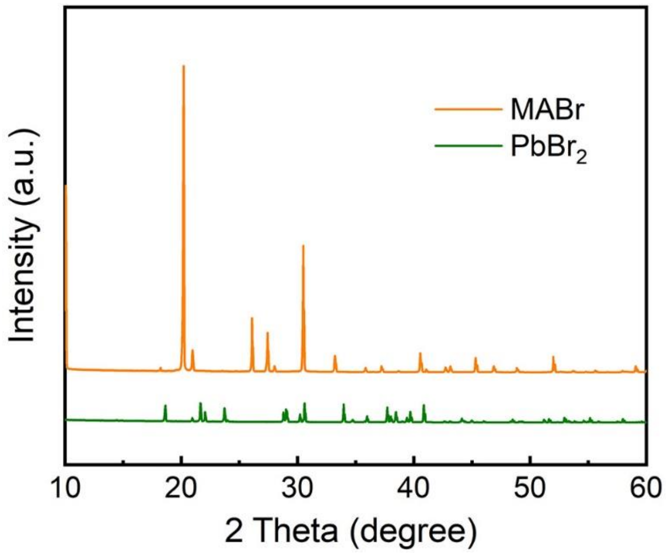

Figure S16. XRD patterns of raw materials (precursor powders: MABr, $PbBr_2$) for the growth of $MAPbBr_3$ SCTFs.

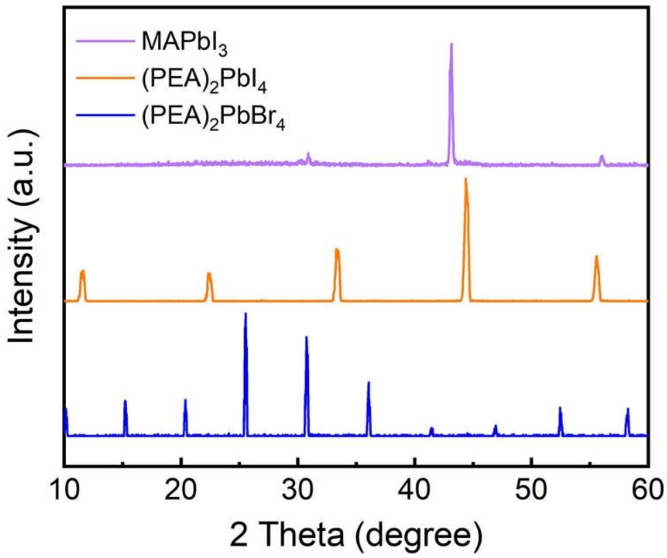

Figure S17. XRD patterns of $MAPbI_3$, $(PEA)_2PbI_4$, $(PEA)_2PbBr_4$ thin films grown on poly-TPD at 2 °C/h. The peaks in XRD suggests high-quality, SCTFs can be grown using the method in this work, which is also applicable to different materials in the perovskite family.

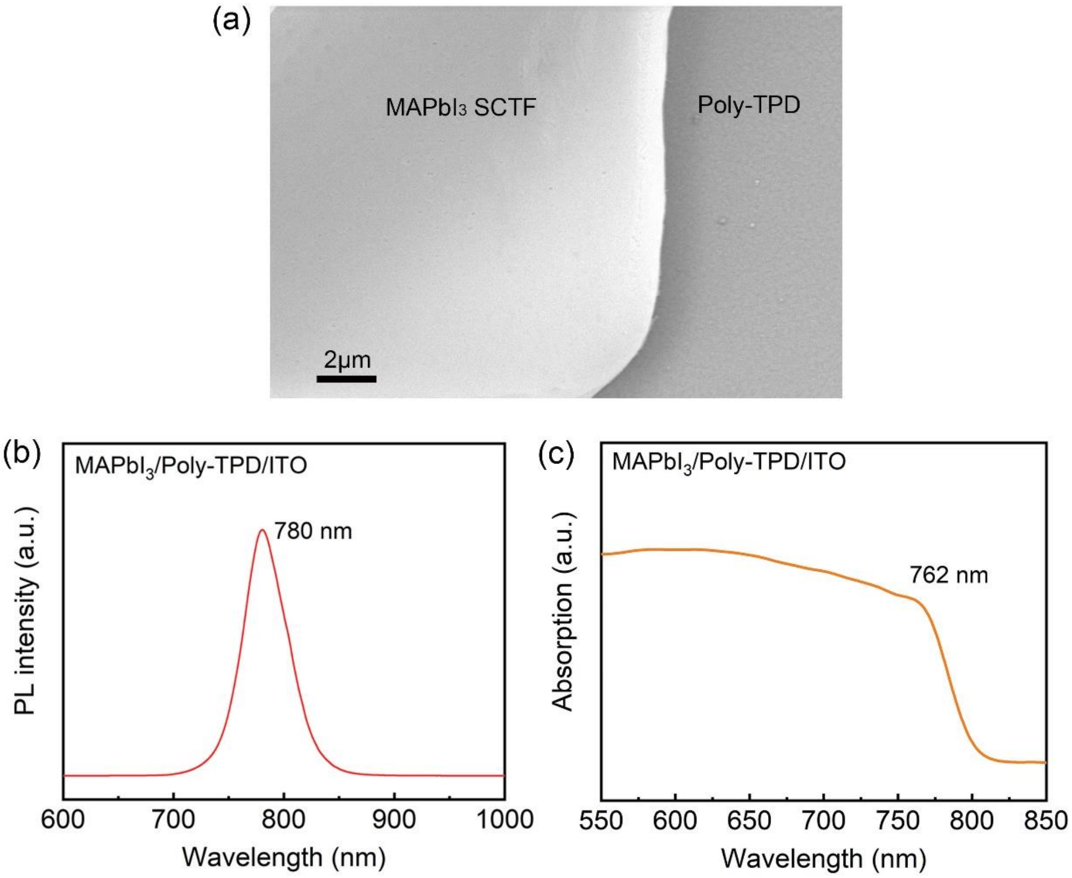

Figure S18. SEM image (a), PL spectrum (b) and absorption spectrum (c) for a single $MAPbI_3$ SCTF on a Poly-TPD/ITO/glass substrate.

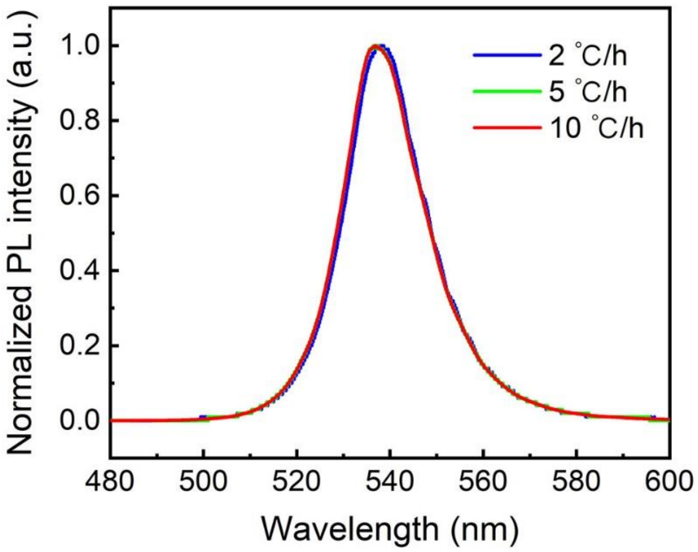

Figure S19. PL for $MAPbBr_3$ SCTFs grown on Poly-TPD at different heating rates.

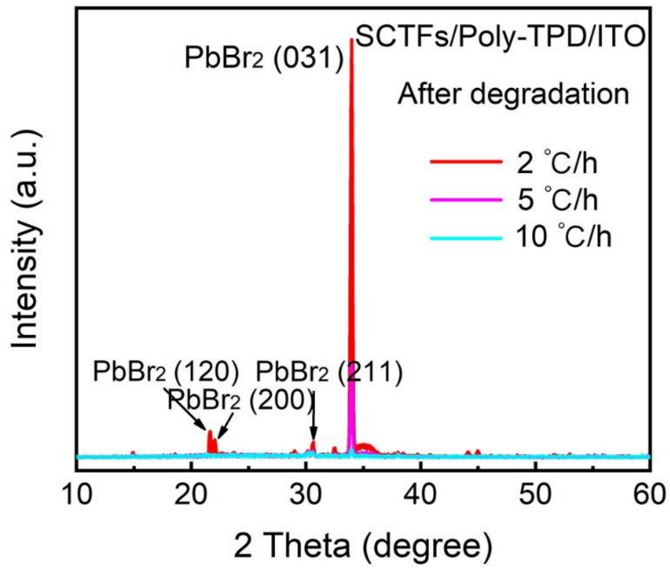

Figure S20. XRD patterns of degradated product of $MAPbBr_3$ SCTFs grown on Poly-TPD at different heating rates, showing the main degradated product is $PbBr_2$. The Miller indices of $PbBr_2$ are from PDF#031-0679.

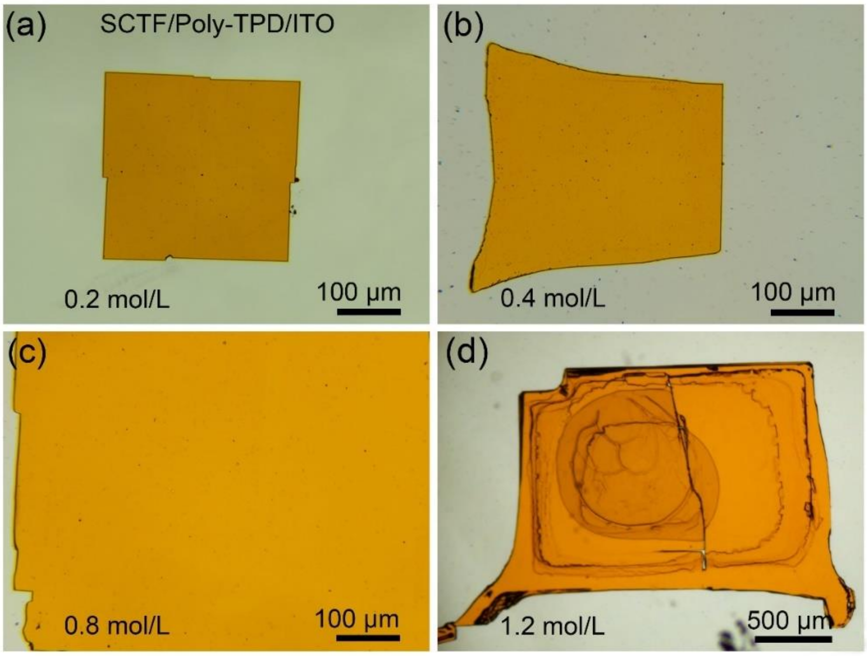

Figure S21. Optical images of the SCTFs prepared with precursor concentrations of (a) 0.2, (b) 0.4, (c) 0.8, and (d) 1.2 mol/L under a heating rate of 2 °C/h and dead load of 2 kg.

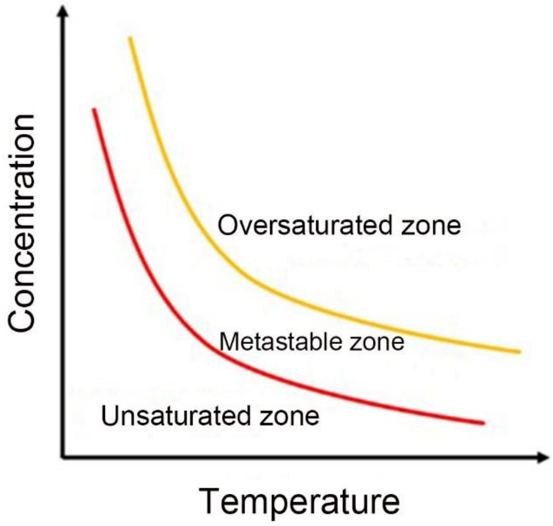

Figure S22. Schematic of concentration vs. temperature.

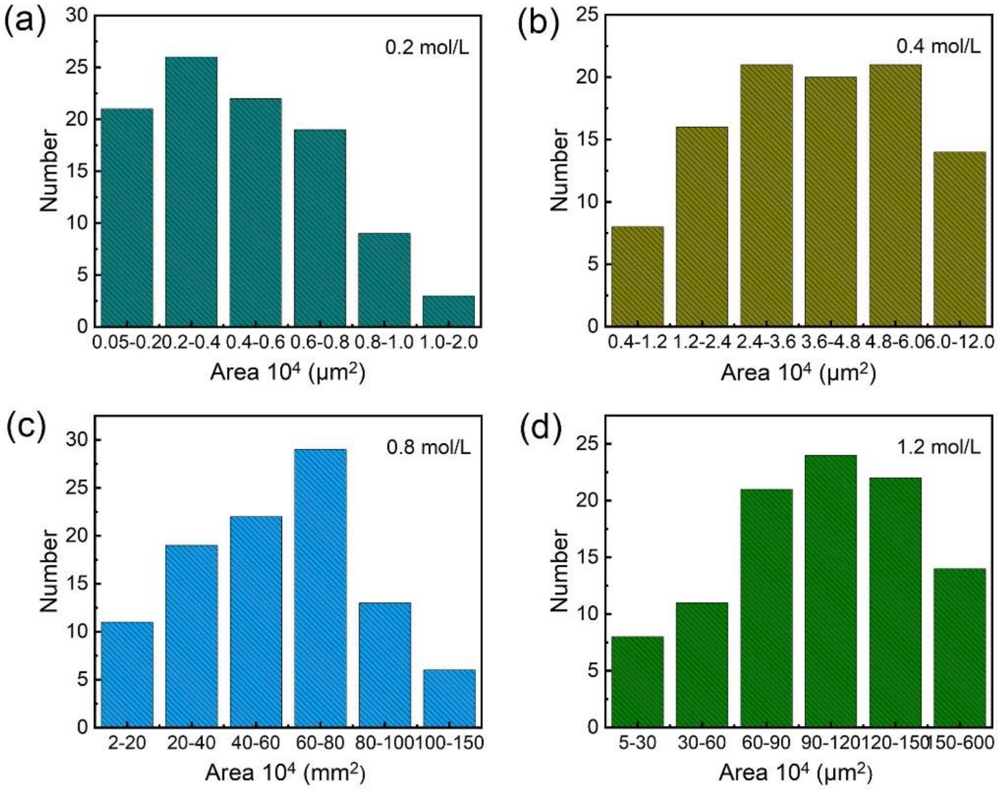

Figure S23. Size distribution of $MAPbBr_3$ SCTFs grown with different precursor concentrations: (a) 0.2 mol/L, (b) 0.4 mol/L, (c) 0.8 mol/L and (d) 1.2 mol/L.

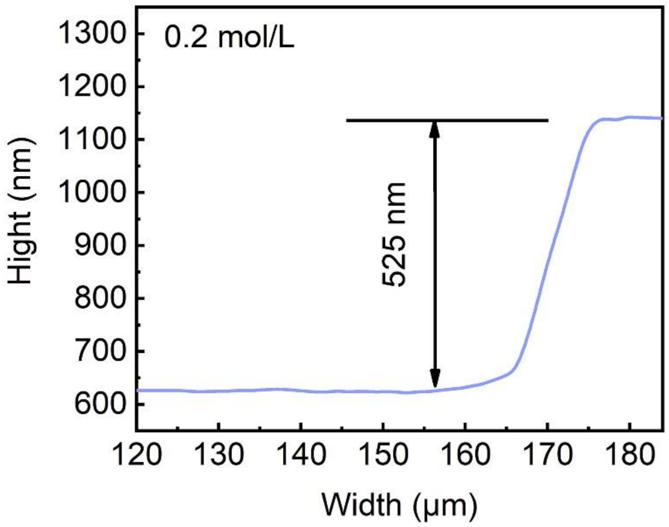

Figure S24. Thickness of $MAPbBr_3$ SCTFs synthesized with concentration of 0.2 mol/L.

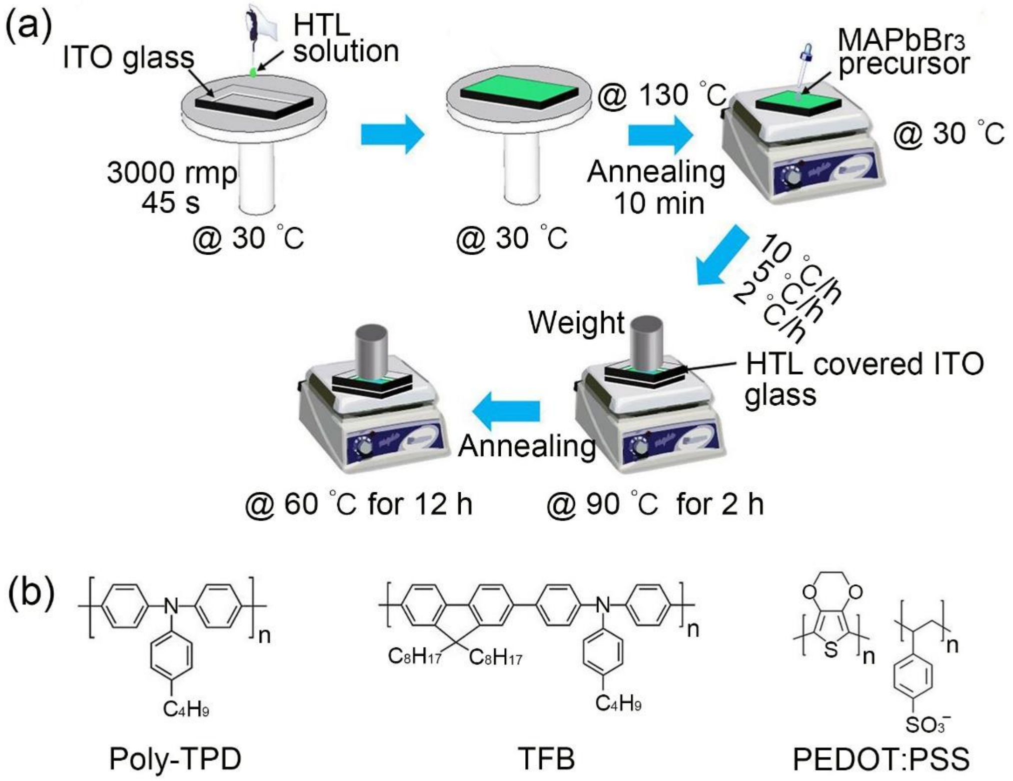

Figure S25. (a) Schematic for the fabrication of a layer of $MAPbBr_3$ SCTF on a solid substrate, and (b) chemical structures of Poly-TPD, TFB and PEDOT:PSS.

Table S1. Contact angles between the liquids with known polar and dispersive components of surface tension and Poly-TPD

Poly-TPD								
Solvent (L)	Surface tension (mN/m)			Contact angle θ (°)	x	y	k	y_o
	Total σ^{tot}	Disperse σ^{d}	Polar σ^{p}					
Water	72.80[a]	21.80[a]	51.00[a]	99.2	1.53	6.55	-0.51	7.33
DMSO	42.80[a]	27.00[a]	15.80[a]	46.75	0.76	6.94		

[a]Data were directly obtained from literature[1].

Here, $x = \sqrt{\frac{\sigma_{lv}^p}{\sigma_{lv}^d}}$, $y = \frac{\sigma_{lv}(\cos\theta + 1)}{2\sqrt{\sigma_{lv}^d}}$, $k = \sqrt{\sigma_{sv}^p}$, $y_0 = \sqrt{\sigma_{sv}^d}$. $\sigma_{sv} = \sigma_{sv}^p + \sigma_{sv}^d$. σ_{sl}, σ_{sv} and σ_{lv} are the interface energy between the substrate and the liquid, the surface energy of the solid substrate and the surface energy of the liquid, respectively. The superscripts of "*d*", "*p*" and "tot" represent dispersive, polar components and total of the surface energy, respectively.[1–4] $\sigma^{tot} = \sigma^p + \sigma^d$. σ_{sv}^p and σ_{sv}^d can be obtained by the linear function[1] $y = kx + y_o$ given by Young's equation.[4]

The interface energies (σ_{sl}) between the substrate and perovskite precursor solution are calculated using the Young's equation[1,4] from the surface energy of substrate (σ_{sv}), surface tension of precursor solution (σ_{lv}) and contact angle between the two phases (θ):

$$\cos\theta = \frac{\sigma_{sv} - \sigma_{sl}}{\sigma_{lv}}$$

Table S2. Contact angles between the liquids with known polar and dispersive components of surface tension and TFB

TFB								
Solvent (L)	Surface Tension (mN/m)			Contact angle $\boldsymbol{\theta}$ (°)	$\boldsymbol{X}$	$\boldsymbol{y}$	$\boldsymbol{k}$	$\mathbf{y_o}$
	Total σ^{tot}	Disperse σ^{d}	Polar σ^{p}					
Water	72.80[a]	21.80[a]	51.00[a]	97.05	1.53	6.84	-0.03	6.89
DMSO	42.80[a]	27.00[a]	15.80[a]	48.35	0.76	6.86		

[a]Data were directly obtained from literature.[1]

Table S3. Contact angles between the liquids with known polar and dispersive components of surface tension and PEDOT:PSS

PEDOT:PSS								
Solvent (L)	Surface Tension (mN/m)			Contact angle $\boldsymbol{\theta}$ (°)	$\boldsymbol{X}$	$\boldsymbol{y}$	$\boldsymbol{k}$	$\mathbf{y_o}$
	Total σ^{tot}	Disperse σ^{d}	Polar σ^{p}					
Water	72.80[a]	21.80[a]	51.00[a]	15.25	1.53	15.32	9.47	0.83
DMSO	42.80[a]	27.00[a]	15.80[a]	18.35	0.76	8.03		

[a]Data were directly obtained from literature.[1]

Table S4. Interface energies between the precursor solution and substrates

	σ_{sv} (mN/m)	σ_{lv} (mN/m)	θ (°)	cosθ	σ_{sl} (mN/m)
NiO_x	81.79	35.47	10.69	0.98	46.94
ZnMgO	69.67	35.47	16.92	0.96	35.74
PTAA	21.96	35.47	53.93	0.59	1.08
SnO_2	90.05	35.47	12.32	0.98	55.40

Table S5. Fitting data from the decay curves of SCTFs/Poly-TPD

Samples (SCTFs/Poly-TPD)	A_1	τ_1	A_2	τ_2
10 ℃/h	0.696	23.751 ns	0.304	5.313 ns
5 ℃/h	0.840	49.398 ns	0.160	7.533 ns
2 ℃/h	0.614	2504 ns	0.386	36.879 ns

Table S6. Fraction of elements in SCTFs/Poly-TPD

Element	Line Type	Weight %	Weight % (Sigma)	Atomic %
Pb	M	48.80	0.39	26.88
Br	L	51.20	0.39	73.12
Total		100.00		100.00

References

[1] A. A. Zhumekenov, V. M. Burlakov, M. I. Saidaminov, A. Alofi, M. A. Haque, B. Turedi, B. Davaasuren, I. Dursun, N. Cho, A. M. El-Zohry, M. De Bastiani, A. Giugni, B. Torre, E. Di Fabrizio, O. F. Mohammed, A. Rothenberger, T. Wu, A. Goriely, O. M. Bakr, *ACS Energy Lett.* **2017**, *2*, 1782.

[2] B. Jańczuk, T. Białlopiotrowicz, *J. Colloid Interface Sci.* **1989**, *127*, 189.

[3] D. K. Owens, R. C. Wendt, *J. Appl. Polym. Sci.* **1969**, *13*, 1741.

[4] T. Young, *Philos. Trans. R. Soc. London* **1805**, *95*, 65.
